# Genetic Profiling of Sodium Channels in Diabetic Painful and Painless and Idiopathic Painful and Painless Neuropathies

**DOI:** 10.3390/ijms24098278

**Published:** 2023-05-05

**Authors:** Rowida Almomani, Maurice Sopacua, Margherita Marchi, Milena Ślęczkowska, Patrick Lindsey, Bianca T. A. de Greef, Janneke G. J. Hoeijmakers, Erika Salvi, Ingemar S. J. Merkies, Maryam Ferdousi, Rayaz A. Malik, Dan Ziegler, Kasper W. J. Derks, Gidon Boenhof, Filippo Martinelli-Boneschi, Daniele Cazzato, Raffaella Lombardi, Sulayman Dib-Hajj, Stephen G. Waxman, Hubert J. M. Smeets, Monique M. Gerrits, Catharina G. Faber, Giuseppe Lauria, on behalf of the PROPANE Study Group

**Affiliations:** 1 Department of Medical Laboratory Sciences, Jordan University of Science and Technology, Irbid 22110, Jordan; 2 Clinical Genomics Unit, Department of Genetics and Cell Biology, Maastricht University, 6229 ER Maastricht, The Netherlands; 3 Department of Neurology, School of Mental Health and Neuroscience, Maastricht University Medical Centre+, 6229 HX Maastricht, The Netherlands; 4 Neuroalgology Unit, IRCCS Foundation “Carlo Besta” Neurological Institute, 20133 Milan, Italy; 5 Department of Toxicogenomics, Maastricht University, 6229 ER Maastricht, The Netherlands; 6 Department of Neurology, Curaçao Medical Center, 4365+37Q, J. H. J. Hamelbergweg, Willemstad, Curacao; 7 Institute of Cardiovascular Sciences, Manchester University NHS Foundation Trust, Manchester Academic Health Science Centre, Manchester M13 9P, UK; 8 Weill Cornell Medicine-Qatar, Doha P.O. Box 24144, Qatar; 9 German Diabetes Centre, 40225 Düsseldorf, Germany; 10 Department of Clinical Genetics, Maastricht University Medical Centre+, 6229 HX Maastricht, The Netherlands; 11 Institute for Clinical Diabetology, German Diabetes Center, Leibniz Center for Diabetes Research, 40225 Düsseldorf, Germany; 12 Laboratory of Human Genetics of Neurological Disorders, Institute of Experimental Neurology (INSPE), Division of Neuroscience, San Raffaele Scientific Institute, 20132 Milan, Italy; 13 Department of Neurology, Yale University School of Medicine, New Haven, CT 06510, USA; 14 Department of Biomedical and Clinical Sciences “Luigi Sacco”, University of Milan, 20157 Milan, Italy

**Keywords:** diabetic neuropathy, small fiber neuropathy, neuropathic pain, molecular inversion probes, next generation sequencing, sodium channel genes variants

## Abstract

Neuropathic pain is a frequent feature of diabetic peripheral neuropathy (DPN) and small fiber neuropathy (SFN). Resolving the genetic architecture of these painful neuropathies will lead to better disease management strategies, counselling and intervention. Our aims were to profile ten sodium channel genes (SCG) expressed in a nociceptive pathway in painful and painless DPN and painful and painless SFN patients, and to provide a perspective for clinicians who assess patients with painful peripheral neuropathy. Between June 2014 and September 2016, 1125 patients with painful-DPN (*n* = 237), painless-DPN (*n* = 309), painful-SFN (*n* = 547) and painless-SFN (*n* = 32), recruited in four different centers, were analyzed for *SCN3A, SCN7A-SCN11A* and *SCN1B-SCN4B* variants by single molecule Molecular inversion probes-Next Generation Sequence. Patients were grouped based on phenotype and the presence of SCG variants. Screening of *SCN3A, SCN7A-SCN11A,* and *SCN1B-SCN4B* revealed 125 different (potential) pathogenic variants in 194 patients (17.2%, *n* = 194/1125). A potential pathogenic variant was present in 18.1% (*n* = 142/784) of painful neuropathy patients vs. 15.2% (*n* = 52/341) of painless neuropathy patients (17.3% (*n* = 41/237) for painful-DPN patients, 14.9% (*n* = 46/309) for painless-DPN patients, 18.5% (*n* = 101/547) for painful-SFN patients, and 18.8% (*n* = 6/32) for painless-SFN patients). Of the variants detected, 70% were in *SCN7A, SCN9A, SCN10A* and *SCN11A*. The frequency of *SCN9A* and *SCN11A* variants was the highest in painful-SFN patients, *SCN7A* variants in painful-DPN patients, and *SCN10A* variants in painless-DPN patients. Our findings suggest that rare SCG genetic variants may contribute to the development of painful neuropathy. Genetic profiling and SCG variant identification should aid in a better understanding of the genetic variability in patients with painful and painless neuropathy, and may lead to better risk stratification and the development of more targeted and personalized pain treatments.

## 1. Introduction

Neuropathic pain (NeuP) arises due to damage or a disease of the somatosensory nervous system involving peripheral fibers (Aβ, Aδ, and C fibers) and central neurons [1]. NeuP is a frequent feature of peripheral neuropathy that causes a significant impact on patients’ quality of life and health care costs [2]. Not all individuals with neuropathy develop NeuP, and it is not possible to predict who is more or less susceptible among those with similar risk exposure.

In small fiber neuropathy (SFN), thinly myelinated Aδ and the unmyelinated C fibers are affected, resulting in sensory symptoms and autonomic dysfunction [3]. Pure SFN can be diagnosed on clinical symptoms, a reduction in intraepidermal nerve fiber density (IENFD), and/or an abnormal quantitative sensory testing (QST). Diabetes mellitus (DM) and sodium channel gene genetic variants are associated with SFN [4]. Establishing the underlying cause of SFN is important, as some of these conditions can be treated [4].

Diabetic peripheral neuropathy (DPN) is the most commonly reported complication of diabetes, affecting 50–90% of patients [5]. DPN is defined as a symmetrical, length-dependent sensorimotor polyneuropathy, with large and/or small fiber involvement, that develops as a consequence of longstanding hyperglycemia, associated metabolic derangements and cardiovascular risk factors [6]. Clinically, painful-DPN is characterized by burning, tingling or shooting pain which interferes with the daily activities of living and social functioning with a significant decrease in the quality of life [7,8]. The treatment of DPN relies on improved glycemic control and the use of medication to reduce NeuP. However, the currently available treatments for painful DPN are inadequate and are associated with side-effects, which limits efficacy. A better understanding of the pathogenesis of painful DPN is urgently needed.

Pathogenic genetic variants of voltage-gated sodium channel genes have been reported in patients with a painful neuropathy [9,10,11,12,13]. These genes play an essential role in action potential generation in nociceptors and their propagation along the axons [14]. Given the importance of sodium channel genes (SCG) in the activity of nociceptors, the exploration of these genes may extend our knowledge of the pathophysiology of NeuP in DPN and SFN, leading to the identification of new molecular druggable sites, with the possibility of developing more effective, personalized treatment strategies.

Single-molecule Molecular Inversion Probes-Next Generation Sequencing (smMIPs-NGS) has recently been introduced for the re-sequencing of gene panels in large cohorts of patients. Using this relatively inexpensive and reliable technique [15], ten genes encoding the alpha-subunits of Nav 1.3 (*SCN3A*), Nav 1.6 (*SCN8A*), Nav 1.7 (*SCN9A*), Nav 1.8 (*SCN10A*), Nav 1.9 (*SCN11A*), Nax (*SCN7A*) and beta-subunits (*SCN1B, SCN2B, SCN3B and SCN4B*) were sequenced in patients with painful-DPN, painless-DPN, and painful and painless SFN. The aim of the current study was to profile these genes according to the different phenotypes and to provide a perspective for clinicians who assess patients with peripheral neuropathy.

## 2. Results

### 2.1. Patient Characteristics

**Figure 1:**
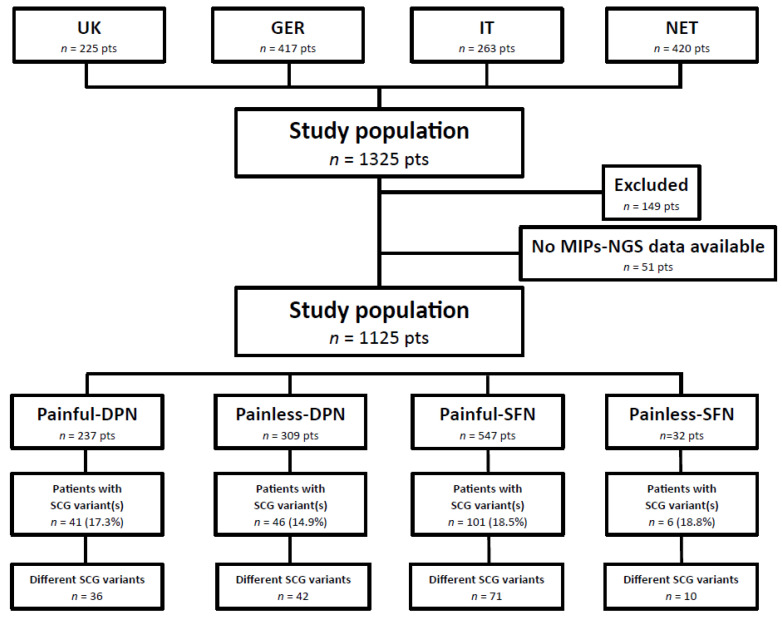
PROPANE study patients analyzed for potentially pathogenic SCG variants by single molecule Molecular Inversion Probe-Next generation sequencing (smMIP-NGS). Between June 2014 and September 2016, patients with painful and painless diabetic peripheral neuropathy (DPN) and idiopathic small fiber neuropathy (SFN), recruited in four different centers, were screened for *SCN3A, SCN7A-SCN11A*, and *SCN1B-SCN4B* variants by smMIP-NGS. The variants’ pathogenicity was classified according to current classification guidelines [16]. UK, University of Manchester, United Kingdom; GER, Deutsche Diabetes Forschungsgesellschaft EV, Germany; IT, Fondazione IRCCS Instituto Neurologico “Carlo Besta”, Italy; NET, Maastricht University Medical Center+, the Netherlands; *n*, number; pts, patients; Painful-DPN, painful-diabetic peripheral neuropathy; Painless-DPN, painless-diabetic peripheral neuropathy; Painful-SFN, painful-idiopathic small fiber neuropathy; Painless-SFN, painless-idiopathic small fiber neuropathy; SCG, sodium channel genes.

A total of 1325 patients were recruited for this study. Two-hundred patients were excluded based on inclusion/exclusion criteria, incomplete clinical or diagnostic data, and/or low quality smMIPs-NGS data (Figure 1). Eventually, 1125 patients, including 237 painful-DPN, 309 painless-DPN, 547 painful-SFN, and 32 painless-SFN patients were profiled for gene variations in *SCN3A, SCN7A-SCN11A*, and *SCN1B-SCN4B*.

Among the 1125 patients, males (55.2%) were represented more than females. The mean age of the patient at recruitment for this study was 58.9 years (SD ± 14.0 years). The median age of onset of complaints was 53.0 years (SD ± 15.4 years) and the mean duration of neuropathy was 7.0 years (SD ± 8.1 years). One in five patients (20.2%) reported a family history of neuropathy.

All patients diagnosed with DPN had an abnormal NCS. In 19.8% of patients the diagnosis of SFN was based on the clinical picture in combination with both an abnormal IENFD and QST, whereas in 26.7% of patients only the IENFD was abnormal, and in 53.5% of patients only the QST was abnormal. Of the DPN patients, 21.2% had type I diabetes, which was more prevalent in painless-DPN compared to painful-DPN (25.6% vs. 15.6%, *p* = 0.005). The patient characteristics per subgroup are given in Table 1.

**Figure 2:**
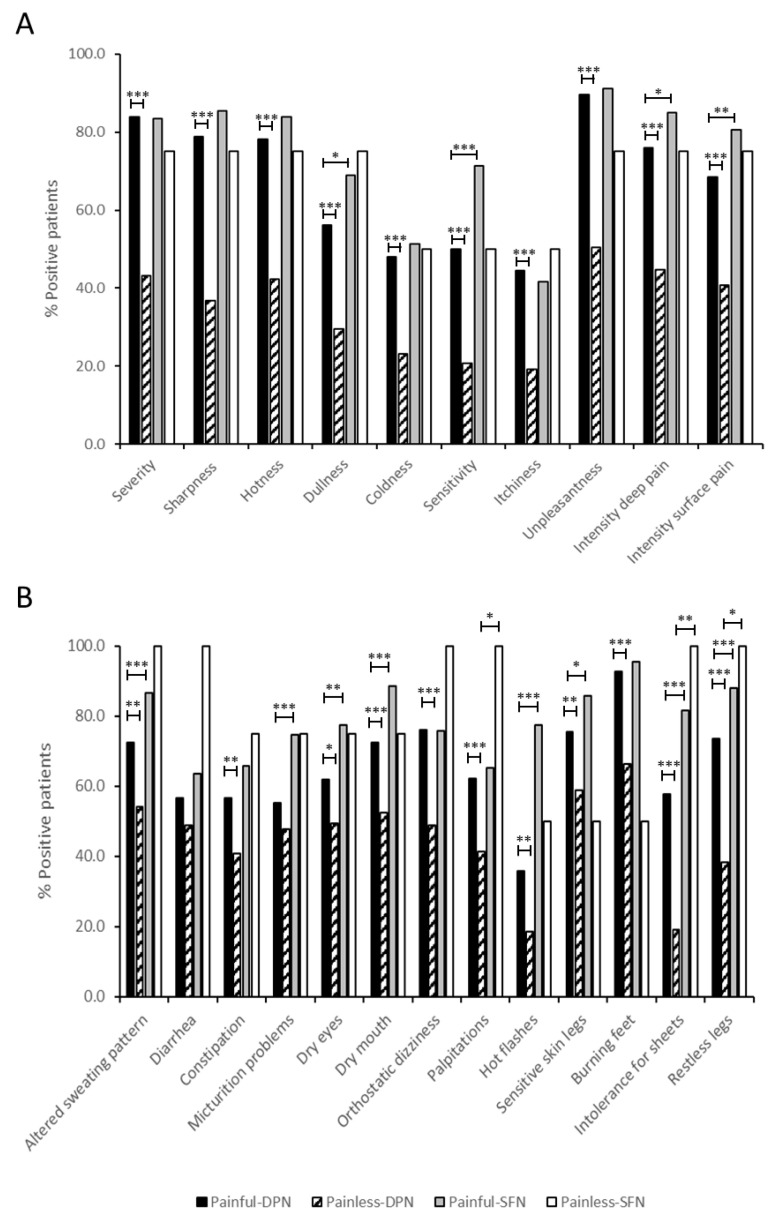
Clinical features in patients with painful and painless diabetic peripheral neuropathy (DPN) and painful and painless idiopathic small fiber neuropathy (SFN). (**A**) Neuropathic Pain Scale (NPS) of painful-DPN (*n* = 149), painless-DPN (*n* = 125), painful-SFN (*n* = 247), and painless-SFN (*n* = 4) patients. Each item is scored on an 11-point scale (0 = not applicable to the experienced pain, and 10 = in the most severe form applicable to the experienced pain). An NPS score > 3 is considered as a relevant pain quality. (**B**) SFN symptoms inventory questionnaire (SFN-SIQ) of painful-DPN (*n* = 152), painless-DPN (*n* = 172), painful-SFN (*n* = 266) and painless-SFN (*n* = 4) patients. The answer options of the SFN-SIQ include ‘never = 1’,‘sometimes = 2’, ‘often = 3’ and ‘always = 4’. A symptom is considered to be present when the score is >1. Statistically significant differences are shown as * for *p* < 0.05, ** for *p* < 0.01, and *** *p* < 0.001. Painful-DPN, painful-diabetic peripheral neuropathy; Painless-DPN, painless-diabetic peripheral neuropathy; Painful-SFN, painful-idiopathic small fiber neuropathy; Painless-SFN, painless-idiopathic small fiber neuropathy.

The quality of NeuP was assessed by NPS and peripheral neuropathy-related symptoms were evaluated using the SFN-SIQ per subgroup. As expected for the 10 qualities of NPS, there was a clear difference between patients with painful neuropathy and painless neuropathy. Comparing patients with painful-DPN and painful-SFN, more patients experienced dullness, sensitivity, intensity deep pain and intensity surface pain in the latter group, while the other qualities of the NPS scored similarly between both groups (Figure 2A).

For the SFN-SIQ, 12 of the 13 symptoms were more often reported by patients with painful neuropathy compared to painless neuropathy, with only diarrhea being reported equally for both patient groups. Nine peripheral neuropathy related symptoms were reported significantly more often by patients with painful-SFN compared to painful-DPN. Bowel problems, orthostatic dizziness, and burning feet were reported equally in both painful neuropathy groups (Figure 2B).

Figure 2 also contains our NPS and SFN-SIQ observations for painless-SFN patients. They were not formally compared with the other three subgroups, because data of this patient group may have been biased due to the low number of patients that completed the NPS and SFN-SIQ questionnaires (*n* = 4).

### 2.2. Performance of SCG-smMIPs-NGS

To profile our study population for ten SCG variations, a smMIPs-NGS targeted enrichment kit was constructed which contained 320 probes to capture all exons and intron-exon junctions (±20 bp) of ten sodium channel genes, *SCN3A, SCN7A-SCN11A*, and *SCN1B-SCN4B*. Targeted regions enrichment, capture, and library sequencing were performed for all patients with painful and painless DPN and painful and painless idiopathic SFN. To calculate the performance and capture efficiency of the SCG-smMIPs-NGS, only the data of patients with complete clinical and diagnostic evaluation and sufficient smMIPs-NGS data were included (*n* = 1125 patients, Figure 1).

The performance and capture efficiency for the ten genes’ smMIPs-NGS was 99.1% (*n* = 317/320 smMIPs) and, on average, 97.6% of the targeted regions were covered >30×. The average coverage of *SCN3A* = 99.6%, *SCN7A* = 100%, *SCN8A* = 97.6%, *SCN9A* = 98.7%, *SCN10A* = 99.9%, *SCN11A* = 99.9%, *SCN1B* = 93.6%, *SCN2B* = 100%, *SCN3B* = 100%, and *SCN4B* = 86.5%. No sequence reads were obtained for three smMIPs (0.9%, *n* = 3/320). We were not able to capture and sequence the first exon of *SCN1B*, *SCN4B* and *SCN11A* genes by smMIPs-NGS. Furthermore, exon 2 of *SCN3B*, exons 13, 16, 21 and 26 of *SCN8A*, exon 13 of *SCN10A* and exon 14 of *SCN11A* showed high variation in sequencing reads. To provide full coverage for *SCN3A*, *SCN8A-SCN11A*, *SCN1B*, and *SCN4B*, 2321 bp were subsequently tested by Sanger sequencing.

### 2.3. SCG Variants in Painful-DPN Patients

In our cohort of 237 patients with painful-DPN, smMIP-NGS of *SCN3A*, *SCN7A-11A*, and *SCN1B-SCN4B* genes revealed 36 different potentially pathogenic heterozygous variants in 41 patients (17.3%, *n* = 41/237, Figure 1). Twelve variants were previously associated with neuropathic pain [9,10,11,12,17,18], of which six have been reported in patients with painful-DPN [12,13]. Twenty-four variants were novel for neuropathic pain, and were classified as VUS (Supplementary Table S1).

Five variants were found in >1 patient. Two patients were positive for three heterozygous *SCN9A* variants; c.2794A > C, c.2971G > T and c.4612T > C. Cell electrophysiology has shown a gain-of-function of *SCN9A* variants, c.2215A > G, c.2794A > C/c.2971G > T [10] and c.4612T > C [17], *SCN11A* variant c.3473T > C [18], and *SCN2B* variant c.325G > A [12], and DRG neuron hyperexcitability for *SCN10A* variant c.4568G > A [9]. For patients with an SCG variant, co-segregation data was not available.

Twelve (potentially) pathogenic variants detected in our cohort of patients with painful-DPN have been reported in patients with epilepsy with or without neurodevelopment delay, autism spectrum disorder, atrial fibrillation, and sudden unexplained/infant death syndrome [19,20,21,22,23]. None of our patients carrying ≥1 of these SCG variants reported a cardiac disorder. Other neurological complaints were not registered in this study.

### 2.4. SCG Variants in Painless-DPN Patients

Among our cohort of 309 painless-DPN patients, 42 different potentially pathogenic heterozygous SCG variants were detected in 46 patients (14.9%, *n* = 46/309, Figure 1) (Supplementary Table S2).

Seven patients harbored more than one SCG variant. One patient was heterozygous for the pathogenic *SCN9A* variants, c.2794A > C and c.2971G > T, and two patients were heterozygous for the likely pathogenic *SCN10A* variants, c.472T > G and c.2441G > A. Four other patients were heterozygous for the VUSs *SCN7A* c.3461G > A and *SCN10A* c.1489C > T, *SCN8A* c.4748T > C and *SCN10A* c.41G > T, *SCN10A* c.2367C > A and c.4736G > A, and *SCN10A* c.5657C > T and *SCN2B* VUS c.625_626delinsCC, respectively. Co-segregation data was not available for any of the variants.

Eight variants have previously been reported in painful-DPN or painful-SFN; *SCN9A* c.2215A > G variants, c.2794A > C/c.2971G > T and c.3799C > G, *SCN10A* variants, c.41G > T, c.3803G > A and c.4568G > A, and *SCN11A* variant c.4282G > A [9,10,11,13]. Except for *SCN11A* c.4282G > A, these variants have also been seen in patients with Dravet syndrome, Autism, Brugada syndrome, atrial fibrillation, and long QT syndrome [19,21,24,25,26].

Five (potentially) pathogenic, *SCN10A* variants: c.472T > G, c.2441G > A and c.5657C > T, *SCN1B* variant c.457G > A, and *SCN3B* variant c.583G > A, have not been seen in patients with painful-DPN and SFN, but three of them have been tested functionally and reported as disease-causing for atrial fibrillation [22], and three VUSs have been reported in patients with Brugada syndrome, atrial fibrillation, and long QT syndrome [27,28]. Only one patient heterozygous for the *SCN10A* variant c.5657C > T and *SCN2B* variant c.625_626delinsCC reported cardiac-related complaints.

### 2.5. SCG Variants in Painful-SFN Patients

For the 547 painful-SFN patients, 71 different potentially pathogenic heterozygous variants were found in 101 patients (18.5%, *n* = 101/547, Figure 1). Forty-six variants were previously described in patients with a neuropathic pain phenotype, including SFN [9,10,11,13,17,18,25,29,30]. Seven variants have already been published as pathogenic [9,10,11], nine variants as likely pathogenic [9,11,18,25], and 28 variants as VUS for a pain phenotype [11,18,31]. Twenty-seven variants were novel for painful-SFN, and were classified as VUS (Supplementary Table S3).

Twenty variants were seen in >1 patient. Eight patients harbored more than one SCG variant, of which one was heterozygous for *SCN3A* c.5583G > T, *SCN9A* c.2794A > C, c.2971G > T, c.4612T > C, and *SCN11A* c.1560G > T. Two patients were heterozygous for *SCN8A* c.1426A > C and *SCN9A* c.2794A > C, c.2971G > T, and c.4612T > C, one patient was heterozygous for *SCN9A* c.2794A > C, c.2971G > T, and c.4612T > C, three patients were heterozygous for *SCN9A* c.2794A > C and c.2971G > T, one patient was heterozygous for *SCN9A* c.1555G > A, c.2271G > A, and *SCN11A* c.3473T > C, and one patient was heterozygous for *SCN9A* c.3799C > G and *SCN11A* c.1142T > C.

Sixteen variants were tested by voltage- and/or current-clamp electrophysiology, and 13 showed a gain-of-function of Na_v_1.7, Na_v_1.8 or Na_v_1.9. Segregation with the disease was seen for seven variants.

Sixteen (potentially) pathogenic variants detected in our patients with painful-SFN have been reported in patients with other neurological and cardiac disorders such as epilepsy with and without neurodevelopmental delay, autism spectrum disorder, Brugada syndrome, atrial fibrillation, and sudden infant death syndrome [19,20,21,24,25,32]. Two patients carrying *SCN10A* variant c.4568G > A were known to have a cardiac disorder.

### 2.6. SCG Variants in Painless-SFN Patients

Ten different potentially pathogenic heterozygous variants were detected in six patients with painless-SFN (18.8%, *n* = 6/32, Figure 1). Eight variants were reported previously in patients with a painful neuropathy [9,10,11,12,17,18].

*SCN3A* c.2077A > G and *SCN4B* c.298C > T were novel for a painful and painless neuropathy, and classified as VUS. Five variants were found in one patient (Supplementary Table S4).

Seven (potentially) pathogenic SCG variants detected in our cohort of patients with painless-SFN have been reported in patients with epilepsy with or without neurodevelopmental delay, autism spectrum disorder, and several cardiac disorders [20,21,24,25,33,34]. None of the patients carrying ≥1 of these SCG variants reported a cardiac disorder.

### 2.7. Shared SCG Variants in Painful and Painless DPN and SFN

In our cohort of 1125 patients with painful and painless DPN and painful and painless SFN, 74 potentially pathogenic variants in 92 patients were specific for a painful neuropathy, and 28 potentially pathogenic variants in 26 patients were specific for a painless neuropathy. Twenty-three potentially pathogenic variants were shared, either between painful-DPN and SFN, or painful-DPN/SFN and painless-DPN. However, *SCN9A* c.2215A > G, c.3799C > G and c.4612T > C, *SCN10A* c.2972C > T, and *SCN11A* c.3473T > C were more often seen in patients with painful neuropathy, while *SCN10A* c.41G > T and c.4568G > A were more often seen in patients with painless neuropathy.

### 2.8. Mutation Frequencies and Distribition of SCG Variants in Patients with Painful and Painless DPN and Painful and Painless SFN

In total, 125 different (potential) pathogenic SCG variants were detected in 194 patients (17.2%, *n* = 194/1125). The overall SCG mutation frequency for painful-DPN patients was 17.3% (*n* = 41/237), for patients with painless-DPN it was 14.9% (*n* = 46/309), for painful-SFN it was 18.5% (*n* = 101/547), and for painless-SFN it was 18.8% (*n* = 6/32).

**Figure 3:**
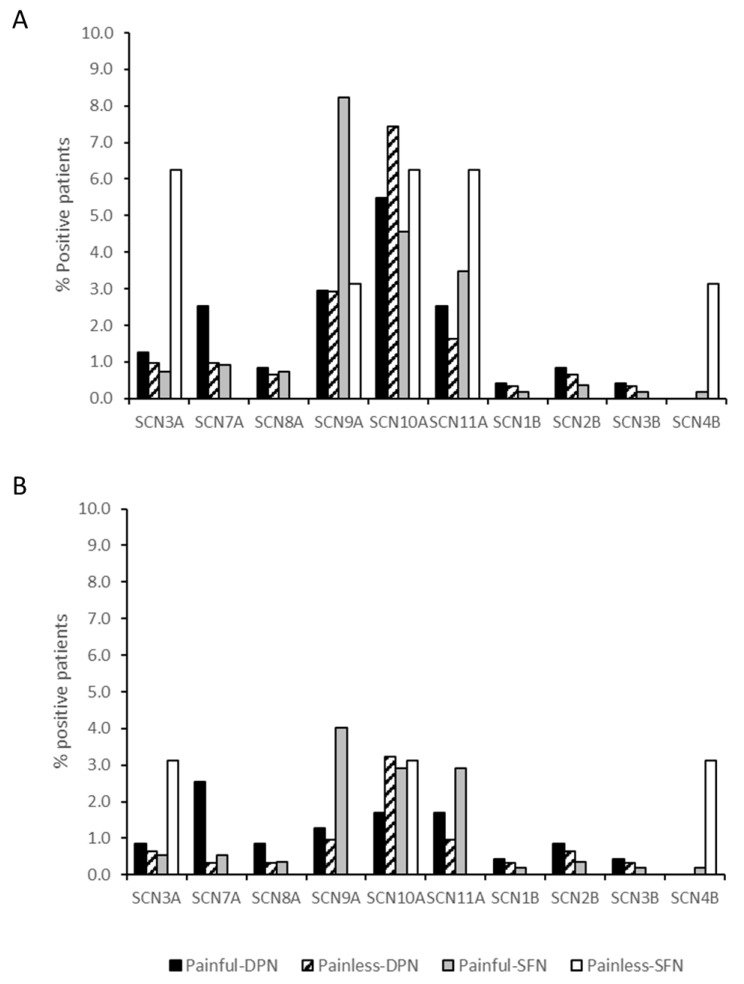
SCG variant positive patients with painful and painless diabetic peripheral neuropathy and painful and painless idiopathic small fiber neuropathy. (**A**) Percentage of *SCN3A*, *SCN7A-SCN11A* and *SCN1B-SFN4B* positive painful-DPN (*n* = 237), painless-DPN (*n* = 309), painful-SFN (*n* = 547) and painless-SFN (*n* = 32) patients with ≥1 variant(s). (**B**) Percentages of *SCN3A*, *SCN7A-SCN11A* and *SCN1B-4B* positive patients with ≥1 variant(s) corrected for variants that were not specific for a painful or painless diabetic/idiopathic neuropathy. Painful-DPN, painful diabetic peripheral neuropathy; Painless-DPN, painless diabetic peripheral neuropathy; Painful-SFN, painful-idiopathic small fiber neuropathy; Painless-SFN, painless-idiopathic small fiber neuropathy; SCG, sodium channel genes.

More than 70% of identified (potential) pathogenic variants were found in *SCN7A, SCN9A, SCN10A* and *SCN11A*. The frequency of *SCN9A* and *SCN11A* variants in patients with painful-SFN was higher than in painful-DPN and painless-DPN, while the frequency of the *SCN10A* variant was lower in patients with painful-DPN and painful-SFN compared to painless-DPN. No significant differences in mutation frequencies were seen for the other tested genes (*SCN3A, SCN8A, SCN1B-SCN4B)* between patients with painful DPN, painless-DPN and painful-SFN (Figure 3A). Data from the patients with painless-SFN was excluded from analysis due to the low number of patients included in the study.

The majority of *SCN9A* variants identified in our cohort of patients with painful SFN were clustered in transmembrane domains I (DI) and II (DII), and in the intracellular linker between DI and DII (Supplementary Figure S2A). The distribution of *SCN9A* variants in patients with painful-DPN was all across the gene (Supplementary Figure S2B), while in patients with painless-DPN they were more localized to DII and domain III (DIII) (Supplementary Figure S2C).

For *SCN10A* variants, the majority of variants were localized to DIII and domain IV (DIV), the intracellular linker between DIII-DIV and the C-terminus of the protein in the patients with painful-SFN (Supplementary Figure S2D). In the patients with painless-DPN, the identified variants were localized to DI and DII (Supplementary Figure S2E). For patients with painful-DPN, no hotspot region for *SCN10A* variants could be identified.

When the above data was corrected for variants that were not specific for a painful or painless phenotype, the overall mutation frequency was 11.8% (*n* = 28/237) for patients with painful-DPN, 7.4% (*n* = 23/309) for patients with painless-DPN, 11.7% (*n* = 64/547) for patients with painful-SFN, and 9.4% (*n* = 3/32) for patients with painless-SFN. For patients with painful-DPN and painful-SFN, approximately 80% of the (potentially) pathogenic variants were identified in *SCN7A, SCN9A, SCN10A,* and *SCN11A,* while for patients with painless-DPN, most variants were identified in *SCN10A* (Figure 3B).

The localization of *SCN9A* variants detected in the patients with painful-SFN and painless-DPN did not change after the exclusion of shared SCG variants. For painful-DPN, only three variants were unique for a painful phenotype. One was localized in the *n*-terminus, and the other two were localized in the linker between DI and DII. For *SCN10A*, the majority of the variants identified in the patients with painful-SFN were localized to domain IV of the protein. No localization of *SCN10A* variants to specific domains was seen in patients with painful-DPN and painless-DPN. For *SCN1*1A, most variants were unique for patients with painful-SFN. The distribution of these variants was all across the gene. For the other tested SCG, the mutation frequency was too low to identify possible hotspot regions.

### 2.9. Clinical Features of Painful-DPN, Painless-DPN, Painful-SFN, and Painless-SFN Patients with and without SCG Variants

To provide a rationale for genetic profiling in patients with painful or painless DPN or painful or painless SFN, clinical features of the four different subgroups with and without SCG variants were compared. Except family history in patients with painful-DPN with and without SCG variants (36.0% vs. 15.3%, *p* = 0.023), no significant differences were seen for any of the patient characteristics for any of the subgroups.

When the comparison was corrected for variants that were not specific for a painful or painless phenotype, and the main patient characteristics were analyzed for all patients with or without SCG variants regardless of their phenotype, the patients with SCG variants more often reported a family history of neuropathy (33.3% vs. 18.8%, *p* = 0.008).

Patients with and without SCG variants were grouped into those with painful or painless neuropathy. In this analysis, the occurrence of coldness and hot flashes was significantly higher in patients with painful neuropathy and SCG variants versus patients with painful neuropathy without SCG variants (coldness, 65.1% vs. 48.1%, *p* = 0.036); hot flashes (77.1% vs. 60.3%, *p* = 0.024). The data was further analyzed according to the presence or absence of SCG variants. The duration of neuropathy was shorter for patients with painful-DPN with SCG variants than for patients with painful-DPN without SCG variants (3.19 [SD ± 2.34] vs. 7.07 [SD ± 6.00), *p* < 0.001), while dry eyes were reported more often in patients with painful-SFN and painless-DPN with SCG variants than patients with painful-SFN and painless-DPN without SCG variants (painful-SFN, 91.2% vs. 75.0%, *p* = 0.036; painless DPN 87.5% vs. 47.6%, *p* = 0.033). All features analyzed after correction for non-specific painful or painless SCG variants are shown in Supplementary Table S5.

## 3. Discussion

There is a strong body of evidence implicating sodium channel genes in human painful disorders [35]. Gain-of-function mutations in *SCN9A*, *SCN10A*, and *SCN11A* genes are associated with painful neuropathy as a consequence of dorsal root ganglion (DRG) neuron hyperexcitability. The pathogenic role of mutations in *SCN9A*, *SCN10A*, and *SCN11A* genes has triggered substantial interest in other sodium channel genes such as *SCN3A*, *SCN7A*, *SCN8A*, and *SCN1B-SCN4B.* To date, this is the first study that has investigated the presence of genetic variants in ten SCG (*SCN3A*, *SCN7A-SCN11A*, and *SCN1B-SCN4B*) in a large cohort of painful and painless diabetic peripheral neuropathy (DPN) and painful and painless idiopathic small fiber neuropathy (SFN).

Since no validation definition of painless or non-painful neuropathy has been established in the literature to date, in this prospective study we defined a ‘non-painful neuropathy’ as a neuropathy with a mean pain intensity <4 without analgesic drugs on PI-NRS. This cut-off of PI-NRS <4 was chosen because patients with a PI-NRS of 0 to 3 generally do not request treatment with analgesic drugs in clinical practice. Others may disagree with this definition and classify painless/non-painful neuropathy patients differently. If patients were incorrectly allocated as non-painful based on our definition of non-painful neuropathy, there is a potential risk that the interpretation of our clinical data and mutation frequencies per subgroup are biased. However, we assume that the risk is low, since the mean PI-NRS of the painless-DPN patients (*n* = 309) and painless-SFN patients (*n* = 32) were respectively 0.13 (SD ± 0.49) and 0.40 (SD ± 0.93). Of greater concern is the low number of painless-SFN patients enrolled in this study. During the inclusion period, only 32 patients with painless-SFN were seen in one of the participating centers. These patients most likely did not visit our clinics, because they do not request a diagnosis of idiopathic SFN and/or treatment of SFN-related symptoms. Due to this low number of painless-SFN patients, a comparison of clinical data of this patient group with the other 3 subgroups is unreliable and has not been performed. Data from painless-SFN patients were presented in this study only to exclude SCG variants not specific for a painful or painless neuropathy phenotype from the mutation frequencies and variant location analysis.

In our cohort of 1125 patients, we identified 125 different (potential) pathogenic *SCN3A*, *SCN7A-SCN11A*, and *SCN1B-SCN4B* variants in 194 patients (17.2%, *n* = 194/1125). Of the patients with painful neuropathy, 18.1% (*n* = 142/784) were heterozygous for ≥1 SCG variant, versus 15.2% (*n* = 52/341) of those with painless neuropathy. Ten patients harbored (potentially) pathogenic sodium channel variants in more than one sodium channel gene (painless-DPN *n* = 4, painful-SFN *n* = 5, painless-SFN *n* = 1). Considering the presence of SCG variants, the duration of neuropathy was significantly shorter and dry eyes and hot flashes were significantly more often reported by patients with SCG variants.

For painful-SFN, (potentially) pathogenic *SCN9A* variants were found more frequently than sodium channel variants in other genes, followed by *SCN10A* and *SCN11A* variants. These mutation frequencies for SFN patients were similar to those reported previously [2,4,11,18]. For painful-DPN and painless-DPN, (potential) pathogenic variants were found more frequently in *SCN10A* than in other genes (painful-DPN, 5.5%, *n* = 13/237; painless-DPN, 7.4%, *n* = 23/309), followed by *SCN9A* (painful-DPN, 3.0%, *n* = 7/237; painless-DPN, 2.9%, *n* = 9/309), *SCN7A* (painful-DPN, 2.5%, *n* = 6/237), and *SCN11A* (painful-DPN, 2.5%, *n* = 6/237; painless-DPN, 1.6.%, *n* = 5/309). Less frequent variants (<6 variants) were identified in *SCN3A*, *SCN8A*, and *SCN1B-SCN4B.* The mutation frequencies for *SCN3A*, *SCN8A*, and *SCN1B-SCN4B* did not differ significantly between painful and painless DPN and painful idiopathic SFN.

After correcting our data for variants that were not specific for a painful or painless phenotype, we show that the frequencies of (potentially) pathogenic variants in *SCN9A*, *SCN10A* and *SCN11A* genes were 3.1% (painful-DPN, 1.3%, *n* = 3/237; painful-SFN, 4.0%, *n* = 22/547), 2.6% (painful-DPN, 1.7%, *n* = 4/237; painful-SFN, 2.9%, *n* = 16/547) and 2.6% (painful-DPN, 1.7%, *n* = 4/237; painful-SFN, 2.9%, *n* = 16/547) respectively, in the patients with painful neuropathy, while they were 0.9% (painful-SFN, 1.0%, *n* = 3/309; painless-SFN, 0%, *n* = 0/32), 3.2% painful-SFN, 3.2%, *n* = 10/309; painless-SFN, 3.1%, *n* = 1/32) and 0.9% (painful-SFN, 1.0%, *n* = 3/309; painless-SFN, 0%, *n* = 0/32), respectively, in the patients with painless neuropathy. Our data contrasts with the study by Wadhawan et al. (2017), who in a cohort of 457 patients showed no significant difference in the missense variant allele frequencies for *SCN9A*, *SCN10A*, and *SCN11A* between patients with painful and non-painful neuropathy [36]. Several rare (potential) pathogenic *SCN9A* variants were identified in patients with both painful-DPN and painless-DPN, whereas Blesneac et al. (2018) had found that 10 out of 111 patients with painful-DPN harbored rare Nav 1.7 variants (potentially pathogenic) compared to none of their patients with painless-DPN (*n* = 78) [13]. These differences may be explained by the use of different criteria to select patients, different populations, and applying different variant filtering strategies.

Shared SCG variants were identified between painful-DPN and painful-SFN, painless-DPN and painless-SFN, as well as painful-DPN/SFN and painless-DPN/SFN. These findings suggest that a single genetic variant may be associated with different phenotypes depending on the presence or absence of other genetic variants in the SCG or other genomic regions and environmental factors. As several of these variants have been reported as gain-of-function mutations [10,17,18], we suggest that these rare genetic variants in the SCG may act as risk factors and/or play a role in predisposing patients to develop a specific phenotype.

A number of studies have shown that rare variants in genes belonging to sodium, calcium, potassium, and transient receptor potential channel gene families are implicated in determining the pain risk threshold and sensitivity [37]; while others have shown that specific polymorphisms in genes, such as *KCNS1*, *SCN9A*, *ADRB2*, *H2TRA*, *CACNG2*, and *IL16* play a role in increasing the risk of chronic pain or an increase in pain sensitivity [2,38,39,40]. There are also reports of genetic variants in, for example, *KCNQ2*, *KCNQ3*, *KNQ3*, *COMT*, *OPRM1*, *TRPV1*, *MC1R*, *GCH1*, and *CACNA2D3* genes that have been associated with pain protection or pain resilience [40,41,42]. Moreover, it appears likely that the development of peripheral neuropathy associated with SCG mutations involves “multi-hit” pathophysiology in which factors such as energetic run-down and changes in transmembrane ionic gradients interact with the presence of the mutation [43,44].

The localization of identified *SCN9A* and *SCN10A* variants in our painful-SFN patient cohort to specific protein domains was similar to that found in previously published data [11]. The clustering of *SCN9A* and *SCN10A* variants was also seen in patients with painless-DPN; however, it was located at different regions of SCN9A and SCN10A in our painful-SFN cohort, in DII and DIII of Nav1.7, and DI and DII of Nav1.8. In our cohort with painful-DPN, no hotspot regions were identified for *SCN9A* and *SCN10A* variants. After exclusion of the non-specific painful and painless SCG variants, the localization of *SCN9A* variants detected in the patients with painful-SFN and painless-DPN did not change. For *SCN10A*, the majority of variants identified in the patients with painful-SFN were localized to DIV, instead of DIII, DIV, the linker between DIII-DIV and C-terminus of the protein, whilst in patients with painless-DPN, the variants were located across the whole gene. Functional studies have shown that SCG variants are associated with both gain-of-function and loss-of-function leading to the impaired inactivation and hyperexcitability or hypoexcitability of neurons [11,39,45,46,47]. Unfortunately, the functional data of variant localization in hotspot regions identified in this study are too limited to correlate channel characteristics with the clinical phenotype.

In our cohort, males were represented more in DPN than SFN, and the patients with DPN were older compared to patients with SFN. There was no difference in the duration of neuropathy between patients with DPN or SFN, although the duration of neuropathy was shorter in patients with painless compared to painful neuropathy. Of note, coldness and hot flashes were experienced more often by patients with painful neuropathy with SCG variants compared to those with painful neuropathy without SCG variants. Also, the unpleasantness of symptoms was higher in patients with painless neuropathy and SCG variants compared to patients with painless neuropathy without SCG variants. Furthermore, a complaint of dry eyes was reported more often in painful-SFN and painless-DPN patients with SCG variants rather than those without SCG variants. The duration of neuropathy was shorter in patients with painful-DPN and SCG variants than in patients with painful-DPN without SCG variants. This finding is in line with the results reported by Blesneac et al., who found that participants carrying rare Nav1.7 variants had been diagnosed for a significantly shorter duration [13]. However, the difference we have seen with the SFN-SIQ for those with painful-SFN contrasts with Eijkenboom et al., 2018, who reported comparable NPS and SFN-SIQ in 1139 pure-SFN patients with and without the (potentially) pathogenic *SCN9A*, *SCN10A* and *SCN11A* variants [11]. This difference could be explained by the smaller cohort size in our study, the testing of more SCG (10 vs. 3), and the correction for variants not specific for a painful or painless neuropathy phenotype.

## 4. Methods

### 4.1. Study Population

In this prospective study, patients (*n* = 1325) were recruited in four different European centers from June 2014 until September 2016. Patients with DPN were recruited for this study at the University of Manchester (United Kingdom) and the Deutsche Diabetes Forschungsgesellschaft EV (Germany), and patients with SFN were recruited at the Fondazione IRCCS Instituto Neurologico “Carlo Besta” (Italy) and the Maastricht University Medical Center+ (The Netherlands). The local medical Ethical Committees of each center approved this study. Informed consent was given by patients to participate in this study.

### 4.2. Inclusion/Exclusion Criteria and Definitions of Painful/Non-Painful Neuropathy

Patients who met the following inclusion criteria were eligible to participate in this study: (1) A diagnosis of sensory neuropathy, based on established clinical and neurological examination, nerve conduction studies (NCS), quantitative sensory testing (QST) and/or skin biopsy findings [6] with diabetic or idiopathic etiology, after ruling out other known causes of neuropathy including vitamin deficiencies, and immune-mediated disorders such as sarcoidosis, Sjögren’s syndrome, celiac disease, leprosy, Epstein-Barr virus, toxins and drugs [4]; (2) Definite or probable NeuP based on the definition and grading system of Treede et al., 2008 [48] for more than one year or no pain; (3) Age of 18 years or older.

DPN patients were included in the study if the diagnosis of DPN was established based on clinically confirmed diabetes, clinical signs of sensory neuropathy, and abnormal NCS, and the absence of the previous mentioned known causes of neuropathy. If NCS was normal (no large fiber involvement), decreased IENFD was required to confirm DPN. Patients without confirmed DPN were excluded from the study.

Idiopathic SFN patients were included in the study if the diagnosis SFN was established based on clinical signs of sensory neuropathy, decreased IENFD on skin biopsy and/or abnormal QST, with no evidence of large nerve fiber damage on neurological examination and NCS, and the absence of the previous mentioned known causes of neuropathy. If IENFD was normal, abnormal QST was required to confirm SFN. Patients without confirmed SFN were excluded from the study.

Patients with pain, categorized as possible NeuP by Treede et al., 2008 [48], or <1 year at screening visit, or non-NeuP were not approached to participate in the study.

As at the start of this study no validated definition of ‘non-painful neuropathy’ was established in the literature, we defined DPN or SFN patients with NeuP by Treede et al., 2008 [48] (possible NeuP excluded) for >1 year and a mean pain intensity ≥ 4 on the pain intensity numerical rating scale (PI-NRS) at the screening visit as ‘painful neuropathy’ patients, and DPN or SFN patients with no pain (PI-NRS, 0) or NeuP by Treede et al., 2008 [48] (possible NeuP excluded) for >1 year and a mean pain intensity < 4 without analgesic drugs on PI-NRS as ‘non-painful neuropathy´ patients, because in clinical practice patients with a PI-NRS <4 generally do not request treatment with analgesic drugs.

### 4.3. Clinical Assessments

After receiving written informed consent, all eligible patients underwent a screening visit and the following records and investigations were undertaken: medical history, including duration of illness, trauma/triggers, age of onset, medication history, and physical activity, family history, neurological examination, nerve conduction studies of the tibial, peroneal and sural nerves, QST for the thermal threshold (Z values ≤ 2.5 = normal and >2.5 = abnormal) at the feet to determine cold, warm, heat-pain and cold-pain thresholds [49], skin biopsy at the distal leg for determination of intra-epidermal nerve fibers density (IENFD), screening for diabetes mellitus, vitamin deficiencies, immune-mediated disorders such as sarcoidosis, Sjögren’s syndrome, celiac disease, leprosy, Epstein-Barr virus, toxins and drugs that cause neuropathy, NeuP pain assessment based on the definition and grading system of Treede et al., 2008 [48], pain intensity on the 11-point PI-NRS (0 = no pain and 10 = worst possible pain) [50], and blood withdrawal (2 × 6 mL EDTA blood) for genetic analyses.

Study participants were then allocated into four groups based on inclusion criteria 1 and 2: painful-DPN, painless-DPN, painful-SFN and painless-SFN, where idiopathic SFN is addressed as ‘SFN’, NeuP (possible NeuP excluded) for >1 year with PI-NRS ≥ 4 as ‘painful’, and no pain/NeuP (possible NeuP excluded) for >1 year with PI-NRS < 4 as ‘painless’ (Supplementary Figure S1).

Subsequently, DNA was isolated from blood (2 × 6 mL EDTA blood) for genetic analyses, and 10 qualities of neuropathic pain were evaluated on the 11-point Neuropathic Pain Scale (NPS; 0 = not applicable to the experienced pain and 10 = in the most severe form applicable to the experienced pain) [51], and 13 SFN-related symptoms were assessed on the 4-point SFN-symptom Inventory questionnaire (SFN-SIQ) scale (1 = never, 2 = sometimes, 3 = often and 4 = always) [52]. An NPS score >3 is considered as a relevant pain quality, and an SFN-related symptom is considered to be present when the SFN-SIQ score is >1.

Except for IENFD, which was optional for DPN patients, all tests were performed for all subgroups.

### 4.4. SCG Mutation Analysis by smMIPs-NGS

Genomic DNA was extracted from whole blood using a QIAamp DNA Blood Maxi Kit/Puregene^®^ Blood Core Kit (Qiagen, Hilden, Germany) or NucleoSpin^®^ 8 Blood Isolation kit (Macherey-Nagel, Düren, Germany) according to the manufacturer’s instructions. Coding exons and exon-flanking intron sequences of *SCN3A*, *SCN7A-SCN11A*, and *SCN1B-SCN4B* were sequenced by smMIP-NGS. Three-hundred-twenty-four smMIPs were designed using a modified version of MIPgen (http://shendurelab.github.io/MIPGEN/ (accessed on 1 August 2014)). The gap-fill length between the extension—and ligation arm (region of interest) of the smMIPs was fixed at 220–230 nt. Probes were synthesized by Integrated DNA Technologies (Coralville, IA, USA).

Targeted capture with smMIPs was performed according to standard protocols [53]. In brief, after hybridization, gap filling and ligation, circularized DNA molecules were used as a template in a PCR with universal primers complementary to the linker sequence. Sample-specific barcode sequences and Illumina adaptors were introduced during the PCR amplification step. Next, samples were pooled and purified using Ampure XP beads according to the manufacturer’s instructions. Pooled samples were sequenced using an Illumina NextSeq500 system (Illumina, Inc., San Diego, CA, USA), with 2 × 150-bp paired-end reads (Illumina, Inc., San Diego, CA, USA). Sequenced data was analyzed by using an in-house smMIPs-NGS data analysis pipeline. Variants were included for analysis with >30× coverage and an alternative variant call of at least 20%. Regions with a poor performance (*n* = 9; coverage <30×, high false positive variant calling) were analyzed by Sanger sequencing [54].

Patients’ coding and immediate flanking regions of *SCN3A*, *SCN7A-SCN11A*, and *SCN1B-SCN4B* were compared with the reference sequence GRCh37. Variants detected were annotated according to the guidelines of the Human Genome Variation Society (http://www.hgvs.org/varnomen/ (accessed on 13 April 2015)), and classified according to current classification guidelines [16] using Alamut Mutation-Interpretation Software (version 2.11, Interactive-Biosoftware, Rouen, France). Variants of interest were confirmed by Sanger sequencing.

### 4.5. Statistical Analysis

The primary analysis was performed to compare patients with painful SFN, painless SFN, painful-DPN, and painless-DPN with and without SCG variants for gender, age, age of onset of neuropathy, duration of neuropathy, familial history of neuropathy, 10 qualities of neuropathic pain evaluated by NPS, and 13 SFN-related symptoms assessed by SFN-SIQ, and to measure the frequencies of the identified SCG variants in the tested patients.

For categorical variables, the ÷2 test was used or the Fisher’s exact test when necessary. For continuous variables, the independent student’s *t*-test was chosen. Equal variances between two groups were tested with the Levene’s test. For these analyses, a significance level of 0.05 was used.

Post hoc analyses were performed to investigate whether differences between patients with specific SCG variants and without variants were present. In total, six post hoc analyses per variable were executed. The analyses were performed in the same way as the primary analyses; however, the significance level was adjusted for multiple testing with the Bonferroni correction.

## 5. Conclusions

In this large cohort of 1125 patients with painful-DPN and painless-DPN and painful-SFN and painless-SFN, 18.1% of patients with painful neuropathy and 15.2% of patients with painless neuropathy carried a potential pathogenic SCG genetic variant. The presence of SCG variants in patients with painful neuropathy was associated with symptoms of coldness and hot flashes. Furthermore, the occurrence of unpleasantness was higher in patients with painless neuropathy with SCG variants.

The phenotypic expression and management of NeuP is undoubtedly complex. SCG variants appear to play a key role in the development of NeuP. As we move forward with applying the genetic testing of SCG, an exclusive wealth of data concerning DNA variations, deeper characterization of rare and common SCG variants, improved classification of disease-associated variants, and understanding of the functional consequences of many of these variants will be reached. Consequently, the utility of genetic screening of SCG for routine clinical use will rise, and possibilities for more effective disease treatment, such as the development of selective sodium channel blockers and optimal therapeutic modulation and pain relief, can be achieved. In addition, identifying the underlying genetic mechanism in families with NeuP has great clinical relevance, will improve genetic counseling, and may result in better clinical management in the respective families. Therefore, the genetic screening of SCG should be considered for patients with painful and painless diabetic peripheral neuropathy and painful and painless idiopathic small fiber neuropathy.

## Figures and Tables

**Table 1:** Patient characteristics of the study population.

		Painful-DPN Patients (*n* = 237)	Painless-DPN Patients (*n* = 309)	Painful-SFN Patients (*n* = 547)	Painless-SFN Patients (*n* = 32)
Male (n)		150/237 (63.3%)	236/309 (76.4%)	220/547 (40.2%)	15/32 (46.9%)
Mean age at recruitment (in years ± SD)		63.70 ± 10.42 no data missing from patients	64.92 ± 11.88 no data missing from patients	53.56 ± 14.09 no data missing from patients	54.69 ± 17.11 no data missing from patients
Mean age of onset neuropathy (in years ± SD)		58.43 ± 11.26 data missing from 69 patients	62.29 ± 11.82 data missing from 128 patients	53.56 ± 14.09 no data missing from patients	45.79 ± 14.16 data missing from 13 patients
Mean duration of neuropathy (in years ± SD)		6.70 ± 5.86 data missing from 70 patients	5.62 ± 6.60 data missing from 140 patients	7.91 ± 9.65 data missing from 218 patients	6.42 ± 6.30 data missing from 13 patients
Familial cases of neuropathy (n)		30/162 (18.5%) data missing from 75 patients	24/185 (13.0%) data missing from 124 patients	80/314 (25.5%) data missing from 233 patients	0/4 (0%) data missing from 28 patients
Normal EMG (n)		0/237 (0%)	0/309 (0%)	547/547 (100%)	32/32 (100%)
Abnormal QST (n)		130/161 (80.2%) * data missing from 76 patients	139/182 (76.4%) * data missing from 127 patients	329/347 (94.8%) data missing from 200 patients	12/12 (100%) data missing from 20 patients
Abnormal IENFD (n)		12/12 (100%) * data missing from 225 patients	N/A *	249/495 (50.3%) data missing from 52 patients	21/29 (72.4%) data missing from 3 patients
Abnormal QST + abnormal IENFD (n)		0/12 (0%) * data missing from 225 patients	N/A *	114/347 (32.9%) data missing from 200 patients	1/12 (8.3%) data missing from 20 patients
PI-NRS (mean ± SD)		7.71 ± 1.71 no data missing from patients	0.13 ± 0.49 no data missing from patients	6.51 ± 2.20 no data missing from patients	0.40 ± 0.93 no data missing from patients
Diabetes mellitus	Type 1 (n) Type 2 (n) Unspecified (n)	37/237 (15.6%) 198/237 (83.5%) 2/237 (0.8%)	79/309 (25.6%) 229/237 (74.1%) 1/309 (0.3%)	N/A	N/A
Hypertension (n)		137/237 (57.8%)	155/309 (50.2%)	78/547 (14.3%)	1/32 (3.1%)
Hypercholesterolemia (n)		113/237 (47.7%)	131/309 (42.4%)	50/547 (9.1%)	0/32 (0%)
Cardiovascular diseases (n)		84/237 (35.4%)	105/309 (34.0%)	33/547 (6.0%)	0/32 (0%)

Painful-DPN, painful-diabetic peripheral neuropathy; Painless-DPN, painless-diabetic peripheral neuropathy; Painful-SFN, painful-idiopathic small fiber neuropathy; Painless-SFN, painless-idiopathic small fiber neuropathy; n, number; SD, standard deviation; N/A, not applicable; *, not required for diagnosis of DPN.

## Data Availability

Not applicable.

## Supplementary Materials

The following supporting information can be downloaded at: https://www.mdpi.com/article/10.3390/ijms24098278/s1.
