# Supplementary material for: Genetic Profiling of Sodium Channels in Diabetic Painful and Painless and Idiopathic Painful and Painless Neuropathies
Source: Int J Mol Sci. 2023 May 5;24(9):8278. doi: 10.3390/ijms24098278 (PMC10179245; doi:10.3390/ijms24098278)
Supplement: Supplementary file 1 [file ijms-24-08278-s001.zip › ijms-2239683-supplementary.pdf]

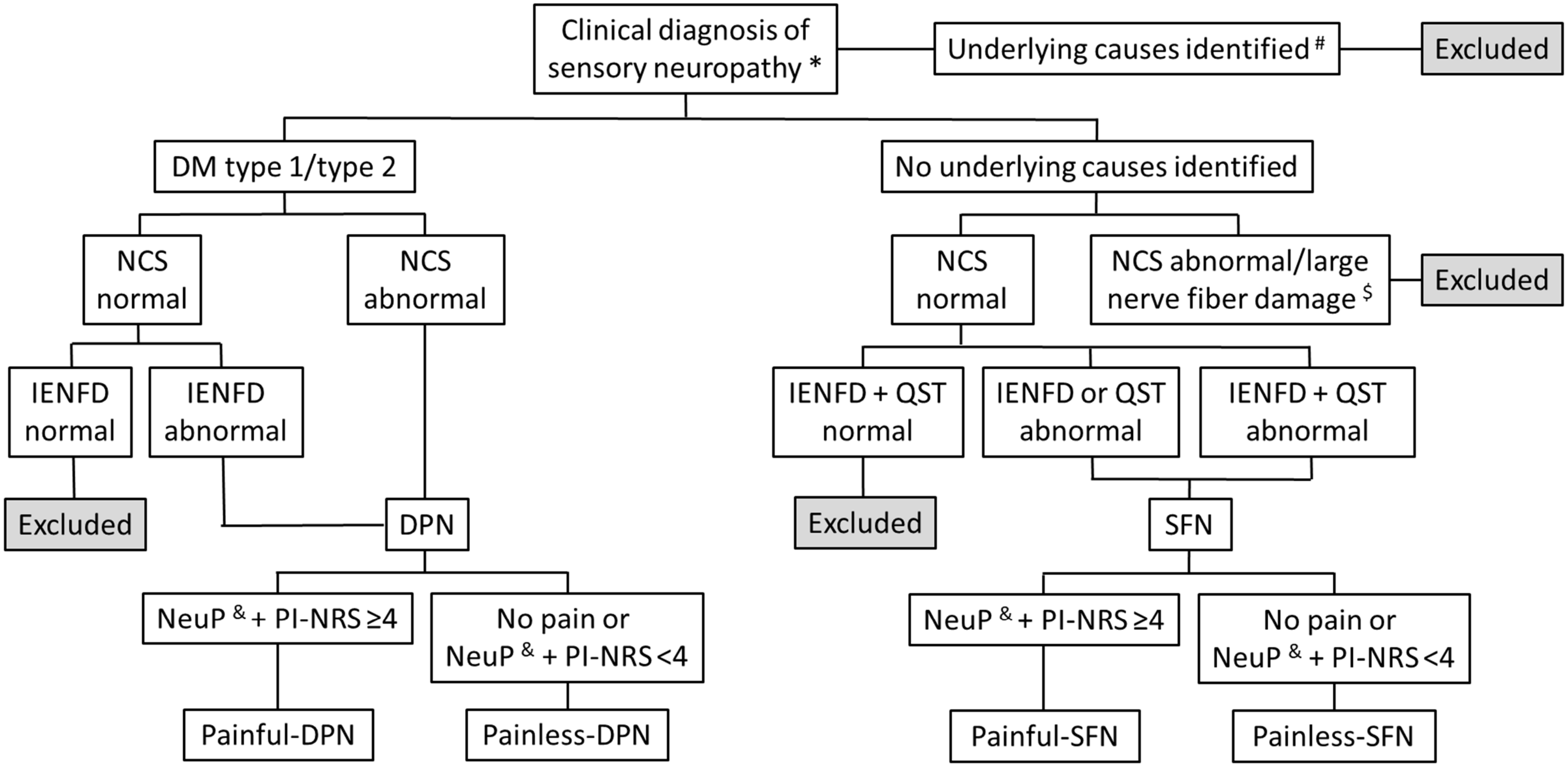

Supplementary Figure S1. Flowchart inclusion PROPANE study patients. DM, diabetes mellitus; NCS, nerve conduction study; IENFD, intra-epidermal nerve fibers density; QST, quantitative sensory testing; DPN, diabetic peripheral neuropathy diabetic; SFN, idiopathic small fiber neuropathy; NeuP, neuropathic pain; PI-NRS, pain intensity numerical rating scale. * Age patient ≥ 18 years, [#] vitamin deficiencies, immune-mediated disorders like sarcoidosis, Sjögren syndrome, coeliac, leprosy, Epstein-Barr virus, toxins and drugs, [$] determined by neurological examination, [&] NeuP assessed by Treede *et al*.,2008 [48] for >1 year (possible NeuP excluded).

Supplementary Table S1. Potentially pathogenic SCG variants (*SCN3A*, *SCN7A-11A*, and *SCN1B-SCN4B*) identified in patients with painful diabetic peripheral neuropathy (painful-DPN, n=237 patients)

Gene	c.position [&]	p.position	Number of patients	Location	MAF gnomAD (%)	Additional variant characteristics	Variant classification	Ref.
SCN3A	c.132T>G	p.(Asp44Glu)	1	N-terminus	0.0021	-	VUS	-
	c.634G>A	p.(Val212Ile)	1	Loop DI/S3-DI/S4	-	-	VUS	-
	c.1619C>T	p.(Ser540Phe)	1	Linker DI/S6-DII/S1	0.15	-	VUS	-
SCN7A	c.538T>A	p.(Trp180Arg)	1	DI/S3	0.0051	-	VUS	-
	c.2612C>T	p.(Ser871Leu)	1	Linker DII/S6-DIII/S1	0.0027	-	VUS	-
	c.2795A>C	p.(Glu932Ala)	1	Linker DII/S6-DIII/S1	0.027	-	VUS	-
	c.3782T>C	p.(Ile1261Thr)	1	DIV/S1	0.0022	-	VUS	-
	c.3791A>G	p.(Gln1264Arg)	1	DIV/S1	-	-	VUS	-
	c.3855G>T	p.(Trp1285Cys)	1	DIV/S2	0.065	-	VUS	-
SCN8A	c.160A>G	p.(lys54Glu)	1	N-terminus	-	-	VUS	-
	c.3529G>A	p.(Glu1177Lys)	1	Linker DII/S6-DIII/S1	0.0012	-	VUS	-
SCN9A	c.185T>C	p.(Ile62Thr)	1	N-terminus	-	-	VUS	-
	c.1208T>C	p.(Met403Thr)	1	Linker DI/S6-DII/S1	0.0046	-	VUS	-
	c.1555G>A	p.(Glu519Lys)	1	Linker DI/S6-DII/S1	0.047	-	VUS	-
	c.2215A>G	p.(Ile739Val)	1	DII/S1	0.25	Gain-of-function Na_v 1.7 channel	VUS (risk factor)	[13]
	c.2794A>C /		2	DII/S6	3.39		Pathogenic	[13]

	c.2971G>T*	p.(Met932Leu) / p.(Val991Leu)		Linker DII/S6-DIII/S1	3.01	Gain-of-function $Na_v1.7$ channel		
	c.3799C>G	p.(Leu1267Val)	1	DIII/S3	0.13	?	VUS	[13]
	c.4612T>C*	p.(Trp1538Arg)	2	Loop DIV/S1-DIV/S2	0.20	Gain-of-function $Na_v1.7$ channel	Pathogenic	[13]
SCN10A	c.1138G>A	p.(Val380Ile)	2	DI/S6	0.058	-	VUS	-
	c.1249_1251del	p.(Lys417del)	2	Linker DI/S6-DII/S1	0.081	-	VUS	-
	c.1492C>T	p.(Arg498Trp)	1	Linker DI/S6-DII/S1	0.0052	-	VUS	‘
	c.2972C>T	p.(Pro991Leu)	3	Linker DII/S6-DIII/S1	0.0.094	-	VUS	
	c.3061C>T	p.(Gln1021*)	1	Linker DII/S6-DIII/S1	0.0012	-	VUS	
	c.3803G>A	p.(Arg1268Gln)	1	Loop DIII/S4-DIII/S5	0.19		VUS	-
	c.3859G>A	p.(Val1287Ile)	1	DIII/S5	0.059	-	VUS	-
	c.4379G>A	p.(Arg1460Gln)	1	Linker DIIIS6-DIV/S1	0.100	-	VUS	-
	c.4568G>A	p.(Cys1523Tyr)	1	DIV/S2	0.11	DRG neuron hyperexcitability	Likely pathogenic	-
SCN11A	c.3473T>C	p.(Leu1158Pro)	3	DIII/S4	0.047	Gain-of-function $Na_v1.9$ channel	Likely pathogenic	-
	c.4282G>A	p.(Gly1428Ser)	1	DIV/S3	0.020		VUS	-
	c.4499T>C	p.(Ile1500Thr)	1	DIV/S5	-	-	VUS	-
	c.4607C>T	p.(Thr1536Ile)	1	Loop DIV/S5-DIV/S6	0.0028	-	VUS	-
SCN1B	c.254G>T	p.(Arg85Leu)	1	β-subunit	-	-	VUS	-
SCN2B	c.319A>C	p.(Lys107Gln)	1	β-subunit	-	-	VUS	-
	c.325G>A	p.(Asp109Asn)	1	β-subunit	0.00071	Gain-of-function $Na_v1.7$ channel	Likely pathogenic	[12]
SCN3B	c.161T>G	p.(Val54Gly)	1	β-subunit	0.0028	-	VUS	-

c.position, location cDNA; p.position, location in protein; MAF gnomAD, Minor Allele Frequency Genome Aggregation Database; VUS, Variants with uncertain clinical significance.

& Variants detected were annotated according to the guidelines of the Human Genome Variation Society using reference sequence GRCh37 and transcript numbers, NM_006922.3 (*SCN3A)*; NM_002976.3 (*SCN7A);* NM_014191.3 (*SCN8A);* NM_002977.3 (*SCN9A)*; NM_006514.2 (*SCN10A)*; NM_014139.2 (*SCN11A)*; NM_001037.4 (*SCN1B)*; NM_004588.4 (*SCN2B)*; NM_018400.3 (*SCN3B).*

* Two patient were heterozygous for *SCN9A* c.2794A>C, c.2971G>T and c.4612T>C.

Supplementary Table S2. Potential pathogenic SCG variants (*SCN3A*, *SCN7A-11A*, and *SCN1B-SCN4B*) identified in patients with painless diabetic peripheral neuropathy (painless-DPN, n=309 patients)

Gene	c.position [&]	p.position	Number of patients	Location	MAF gnomAD (%)	Additional variant characteristics	Variant classification	Ref.
SCN3A	c.1619C>T	p.(Ser540Phe)	1	Linker DI/S6-DII/S1	0.15	-	VUS	-
	c.4679G>A	p.(Arg1560Gln).	1	DIV/S2	0.0011	-	VUS	-
	c.5589G>C	p.(Glu1863Asp)	1	C-terminus	0.0014	-	VUS	-
SCN7A	c.3461C>A*	p.(Ser1154Tyr)	1	Loop DIII/S5-DIII/S6	0.010	-	VUS	-
	c.4072C>T	p.(Arg1358Cys)	1	DIV/S4	0.026	-	VUS	-
	c.4865G>A	p.(Arg1622Gln)	1	C-terminus	0.17	-	VUS	-
SCN8A	c.1475G>A	p.(Arg492His)	1	Linker DI/S6-DII/S1	0.0021	-	VUS	-
	c.4748T>C*	p.(Ile1583Thr)	1	Loop DIV/S2-DIV/S3	0.020	-	VUS	-
SCN9A	c.2215A>G	p.(Ile739Val)	2	DII/S1	0.25	Gain-of-function	VUS (risk factor)	-
	c.2794A>C /<br>c.2971G>T	p.(Met932Leu) /<br>p.(Val991Leu)	1	DII/S6<br>Linker DII/S6-DIII/S1	3.39<br>3.01	Gain-of-function<br>$Na_v1.7$ channel	Pathogenic	-
	c.2819T>C	p.(Val940Ala)	1	Loop DII/S5-DII/S6	0.024	-	VUS	-
	c.2969A>G	p.(Tyr990Cys)	1	Linker DII/S6-DIII/S1	0.060	-	VUS	-
	c.3799C>G	p.(Leu1267Val)	2	DIII/S3	0.13	-	VUS	-
	c.4288A>G	p.(Ile1430Val)	1	Loop DIII/S5-DIII/S6	0.00076	-	VUS	
	c.5458G>T	p.(Val1820Phe).	1	C-terminus	0.0014	-	VUS	-
SCN10A	c.41G>T*	p.(Arg14Leu)	3	N-terminus	0.19	-	VUS	-
	c.368C>T	p.(Ala123Val)	1	N-terminus	0.017	-	VUS	-
	c.472T>G /<br>c.2441G>A	p.(Tyr158Asp) /<br>p.(Arg814His)	2	DI/S2<br>Loop DII/S5-DII/S6	0.026<br>0.031	Gain-of-function<br>$Na_v1.8$ channel	Likely pathogenic	-
	c.1079G>A	p.(Arg360His)	1	Loop DI/S5-DI/S6	0.025	-	VUS	-
	c.1138G>A	p.(Val380Ile)	1	DI/S6	0.058	-	VUS	-
	c.1141A>G	p.(Ile381Val)	2	DI/S6	0.062	-	Likely pathogenic	-
	c.1249_1251del	p.(Lys417del)	1	Linker DI/S6-DII/S1	0.081	-	VUS	-
	c.1489C>T*	p.(Arg497Cys)	1	Linker DI/S6-DII/S1	0.0048	-	VUS	-
	c.2367C>A*	p.(Asn789Lys)	1	Loop DII/S4-DII/S5	0.0021	-	VUS	-
	c.2530C>T	p.(Arg844Cys)	1	Loop DII/S5-DII/S6	0.0032	-	VUS	-
	c.3803G>A	p.(Arg1268Gln)	2	Loop DIII/S4-DIII/S5	0.19	-	VUS	-
	c.3757C>T	p.(Arg1253Cys)	1	DIII/S4	0.0032	-	VUS	-

	c.4568G>A	p.(Cys1523Tyr)	4	DIV/S2	0.11	DRG neuron hyperexcitability	Likely pathogenic	-
	c.4736G>A*	p.(Arg1579Gln)	1	DIV/S4	0.0085	-	VUS	-
	c.5216A>T	p.(Asp1739Val)	1	C-terminus	0.00040	-	VUS	-
	c.5657C>T*	p.(Ala1886Val)	1	C-terminus	0.12	Gain-of-function Na_v1.8 channel	VUS	-
SCN11A	c.712C>T	p.(Arg238Cys)	1	DI/S4	0.019	-	VUS	-
	c.1730C>T	p.(Pro577Leu)	1	DII/S1	0.018	-	VUS	-
	c.2379G>T	p.(Leu793Phe)	1	DII/S6	-	-	VUS	-
	c.4282G>A	p.(Gly1428Ser)	1	DIV/S3	0.020	-	VUS	-
	c.4607C>T	p.(Thr1536Ile)	1	Loop DIV/S5-DIV/S6	0.0028	-	VUS	-
SCN1B	c.457G>A	p.(Asp153Asn)	1	β-subunit	0.0067	-	VUS	-
SCN2B	c.526G>T	p.(Val176Leu)	1	β-subunit	-	-	VUS	-
	c.625_626 delinsCC*	p.(Asn209Pro)	1	β-subunit	-	-	VUS	-
SCN3B	c.583G>A	p.(Ala195Thr)	1	β-subunit	0.0099	-	VUS	-

c.position, location cDNA; p.position, location in protein; MAF gnomAD, Minor Allele Frequency Genome Aggregation Database; VUS, Variants with uncertain clinical significance.

[&] Variants detected were annotated according to the guidelines of the Human Genome Variation Society using reference sequence GRCh37 and transcript numbers, NM_006922.3 (*SCN3A*); NM_002976.3 (*SCN7A);* NM_014191.3 (*SCN8A);* NM_002977.3 (*SCN9A*); NM_006514.2 (*SCN10A*); NM_014139.2 (*SCN11A*); NM_001037.4 (*SCN1B*); NM_004588.4 (*SCN2B)*; NM_018400.3 (*SCN3B).*

* Four patients were respectively heterozygous for *SCN7A* c.3461G>A and *SCN10A* c.1489C>T, *SCN8A* c.4748T>C and *SCN10A* c.41G>T, *SCN10A* c.2367C>A and *SCN10A* c.4736G>A, and *SCN10A* c.5657C>T and *SCN2B* c.625_626delinsCC.

Supplementary Table S3. Potential pathogenic SCG variants (*SCN3A*, *SCN7A-11A*, and *SCN1B-SCN4B*) identified in patients with painful idiopathic small fiber neuropathy (painful-SFN, n=547 patients).

Gene	c.position [&]	p.position	Number of patients	Location	MAF gnomAD (%)	Additional variant characteristics	Variant classification	Ref.
SCN3A	c.2610G>C	p.(Met870Ile)	1	Loop DII/S4-DII/S5	-	-	VUS	-
	c.3072G>C	p.(Glu1024Asp)	1	Linker DII/S6-DIII/S1	-	-	VUS	-
	c.4467_4475delinsCAAAAATT	p.(Gln1489Hisfs*8	1	Linker DIII/S6-DIV/S1	-	-	VUS	
	c.5583G>T*	p.(Leu1861Phe)	1	C-terminus	0.0012		VUS	
SCN7A	c.1501A>C	p.(Ile501Leu)	1	DII/S1	0.01	-	VUS	
	c.2795A>C	p.(Glu932Ala)	1	DIII/S1	0.027	-	VUS	-
	c.3461C>A,	p.(Ser1154Tyr)	1	Loop DIII/S5-DIII/S6	0.010		VUS	-
	c.4072C>T	p.(Arg1358Cys)	1	DIV/S4	0.026		VUS	-
	c.4865G>A	p.(Arg1622Gln)	1	C-terminus	0.17		VUS	-
SCN8A	c.1426A>C*	p.(Ser476Arg)	2	Linker DI/S6-DII/S1	-	-	VUS	-
	c.4719G>A	p.(Met1573Ile)	1	DIV/S2	0.0056	-	VUS	-
	c.4748T>C	p.(Ile1583Thr)	1	DIV/S3	0.020		VUS	-
SCN9A	c.554G>A	p.(Arg185His)	1	Loop DI/S2-DI/S4	0.31	Co-segregation inconclusive, gain-of-function Na_v 1.7 channel	VUS (risk factor)	[9,11]
	c.684C>G	p.(Ile228Met)	4	DI/S4	0.086	Co-segregation with disease, gain-of-function Na_v 1.7 channel	Pathogenic	[9,11]
	c.910T>C	p.(Tyr304His)	1	Loop DI/S5-DI/S6	-	-	VUS	-
	c.1007A>C	p.(Asn336Thr)	1	Loop DI-S5-DI/S6	-		VUS	[11]
	c.1555G>A*	p.(Glu519Lys)	1	Linker DI/S6-DII/S1	0.047		VUS	[11]
	c.1592C>A	p.(Thr531Asn)	1	Linker DI/S6-DII/S1	-	-	VUS	[11]

	c.1859G>A	p.(Ser620Asn)	1	Linker DI/S6-DII/S1	0.00080	-	VUS	-
	c.2033C>A	p.(Ala678Glu)	1	Linker DI/S6-DII/S1	-	-	VUS	[11]
	c.2157G>C	p.(Trp719Cys)	1	Linker DI/S6-DII/S1	0.098	-	VUS	[11]
	c.2159T>A	p.(Ile720Lys)	1	Linker DI/S6-DII/S1	0.016	Co-segregation with disease, gain-of-function Na_v 1.7 channel	Pathogenic	[9,11]
	c.2215A>G	p.(Ile739Val)	7	DII/S1	0.25	Co-segregation inconclusive, gain-of-function Na_v 1.7 channel	VUS (risk factor)	[9,11]
	c.2266C>A	p.(Pro756Thr)	1	Loop DII/S1-DII/S2	0.0029	-	VUS	[11]
	c.2271G>A*	p.(Met757Ile)	1	Loop DII/S1-DII/S2	0.0041	-	VUS	[11]
	c.2567G>A	p.(Gly856Asp)	1	Loop DII/S4-DII/S5	-	Co-segregation with disease, gain-of-function Na_v 1.7 channel	Pathogenic	[9,11]
	c.2759C>A	p.(Thr920Asn)	1	Loop DII/S5-DII/S6	-	-	VUS	-
	c.2794A>C / c.2971G>T*	p.(Met932Leu) / p.(Val991Leu)	7	DII/S6<br>Linker DII/S6-DIII/S1	3.39<br>3.01	Co-segregation with disease, gain-of-function Na_v1.7 channel	Pathogenic	[9,11]
	c.2969A>G	p.(Tyr990Cys)	2	Linker DII/S6-DIII/S1	0.060		VUS	[11]
	c.3689T>C	p.(Met1230Thr)	1	DIII/S2	-	-	VUS	[11]
	c.3799C>G*	p.(Leu1267Val)	9	DIII/S4	0.13	?	VUS	[9,31]
	c.3836G>A	p.(Arg1279Gln)	1	DIII/S4	0.0036	-	VUS	[11]
	c.4612T>C*	p.(Trp1538Arg)	5	DIV/S2	0.20	Co-segregation with disease, gain-of-function Na_v 1.7 channel	Pathogenic	[11,17]
	c.4970A>C	p.(Tyr1657Ser)	1	DIV/S5	-	-	Likely pathogenic	[11]
SCN10A	c.41G>T	p.(Arg14Leu)	2	N-terminus	0.19	Co-segregation inconclusive	VUS	[11]
	c.1138G>A	p.(Val380Ile)	2	DI/S6	0.058	-	VUS	-
	c.1141A>G	p.(Ile381Val)	1	DI/S6	0.062		Likely pathogenic	[11]
	c.2221C>G	p.(Leu741Val)	1	DII/S3	0.0088	-	VUS	[11]

	c.2737G>A	p.(Ala913Thr)	1	Linker DII/S6-DIII/S1	0.033	-	VUS	[11]
	c.2972C>T	p.(Pro991Leu)	4	Linker DII/S6-DIII/S1	0.094	Co-segregation inconclusive (controleren)	VUS	[11]
	c.3482T>C	p.(Met1161Thr)	1	DIII/S1	0.019	-	Likely pathogenic	[11]
	c.3803G>A	p.(Arg1268Gln)	1	Loop DIII/S4-DIII/S5	0.19	-	VUS	[11]
	c.3910G>A	p.(Ala1304Thr)	2	DIII/S5	0.0046	Gain-of-function Na$_v$1.8 channel	Likely pathogenic	[9,11]
	c.4562G>A	p.(Gly1521Asp)	1	DIV/S2	-	-	VUS	[11]
	c.4568G>A	p.(Cys1523Tyr)	3	DIV/S2	0.11	DRG neuron hyperexcitability	Likely pathogenic	[9,11]
	c.4745G>A	p.Arg1582His	1	DIV/S4	-	-	VUS	-
	c.4878G>A	p.(Met1626Ser)	1	DIV/S5	0.0046	-	VUS	[11]
	c.4984G>A	p.(Gly1662Ser)	1	Loop DIV/S5-DIV/S6	0.14	Co-segregation with disease, gain-of-function Na$_v$1.8 channel	Pathogenic	[9,11]
	c.5116A>G	P.(Ile1706Val)	1	DIV/S6	-	Gain-of-function Na$_v$1.8 channel	Likely pathogenic	[11,18]
	c.5263G>A	p.Ala1755Thr	1	C-terminus	0.00080	-	VUS	-
	c.5588G>A	p.(Arg1863Gln)	1	C-terminus	0.0024	-	VUS	-
SCN11A	c.95C>T	p.(Ala32Val)	1	N-terminus	0.063	-	VUS	[11]
	c.1142T>C*	p.(Ile381Thr)	1	DI/S6	0.00080	Gain-of-function Na$_v$1.9 channel	Likely pathogenic	[11,18]
	c.1257G>T	p.(Lys419Asn)	3	Linker DI/S6-DII/S1	0.056	-	VUS	[11,18]
	c.1560G>T*	p.(Gln520His)	1	Linker DI/S6-DII/S1	0.0028	-	VUS	[11]
	c.1744G>A	p.(Ala582Thr)	2	DII/S1	0.015		VUS	[11,18]
	c.2042C>A	p.(Ala681Asp)	1	DII/S4	-	Loss-of-function Na$_v$1.9 channel	VUS	[11,18]
	c.2045A>T	p.(Lys682Ile)	1	DII/S4	-		VUS	-
	c.2095G>A	p.(Gly699Arg)	1	Loop DII/S4-DII/S5	0.016	Gain-of-function Na$_v$1.9 channel	Likely pathogenic	[11,47]
	c.2524G>C	p.(Ala842Pro)	1	Linker DII/S6-DIII/S1	0.0012	-	VUS	[11,18]

	c.3473T>C*	p.(Leu1158Pro)	2	DIII/S4	0.047	Gain-of-function $Na_v1.9$ channel	Likely pathogenic	[11,18]
	c.3506A>G	p.Asn1169Ser	1	Loop DIII/S4-DIII/S5	0.033	-	VUS	-
	c.4049G>A	p.(Arg1350Gln)	1	Linker DIII/S6-DIV/S1	0.0039	-	VUS	[11]
	c.4057-1G>A	p.?	2	Linker DIII/S6-DIV/S1	-	-	VUS	[11,18]
	c.5327G>A	p.(Gly1776Glu	1	C-terminus	-	-	VUS	-
SCN1B	c.632G>A	p.(Cys211Tyr)	1	β-subunit	0.039	-	VUS	-
SCN2B	c.205T>C	p.(Tyr69His)	1	β-subunit	0.0049	DRG neuron hyperexcitability	Likely pathogenic	-
	c.502G>T	p.(Gly168Cys)	1	β-subunit	0.00080	-	VUS	-
SCN3B	c.147G>A	p.(Met49Ile)	1	β-subunit	-	-	VUS	-
SCN4B	c.592_593+1 delAAG	p.(Lys198 Glufs*11)	1	β-subunit	0.0020	-	VUS	-

c.position, location cDNA; p.position, location in protein; MAF gnomAD, Minor Allele Frequency Genome Aggregation Database; VUS, Variants with uncertain clinical significance.

[&] Variants detected were annotated according to the guidelines of the Human Genome Variation Society using reference sequence GRCh37 and transcript numbers, NM_006922.3 (*SCN3A)*; NM_002976.3 (*SCN7A)*, NM_014191.3 (*SCN8A),* NM_002977.3 (*SCN9A)*; NM_006514.2 (*SCN10A)*; NM_014139.2 (*SCN11A)*; NM_001037.4 (*SCN1B)*; NM_004588.4 (*SCN2B)*;, NM_018400.3 (*SCN3B)*; NM_174934.4(*SCN4B).*

* One patient was heterozygous for *SCN3A* c.5583G>T, *SCN9A* c.2794A>C, c.2971G>T, c.4612T>C and *SCN11A* c.1560G>T; two patients were heterozygous for *SCN8A* c.1426A>C and *SCN9A* c.2794A>C, c.2971G>T,c.4612T>C, one patient was heterozygous for *SCN9A* c.2794A>C, c.2971G>T and c.4612T>C, three patients were heterozygous for *SCN9A* c.2794A>C and c.2971G>T, one patient was heterozygous for *SCN9A* c.1555G>A and c.2271G>A and *SCN11A* c.3473T>C, and one patient was heterozygous for *SCN9A* c.3799C>G and *SCN11A* c.1142T>C.

Supplementary Table S4. Potential pathogenic SCG variants (*SCN3A*, *SCN7A-11A*, and *SCN1B-4B*) identified in patients with painless small fiber neuropathy (painless-SFN, n=32 patients).

Gene	c.position [&]	p.position	Number of patients	Location	MAF gnomAD (%)	Additional variant characteristics	Variant classification	Ref.
SCN3A	c.2077A>G	p.(Met693Val)	1	Linker DI/S6-DII/S1	0.032	-	VUS	-
	c.5583G>T*	p.(Leu1861Phe)	1	C-terminus	0.0012		VUS	-
SCN9A	c.2794A>C / c.2971G>T*	p.(Met932Leu) / p.(Val991Leu)	1	DII/S6	3.39	Gain-of-function Na_v1.7 channel	Pathogenic	[9,11]
				Linker DII/S6-DIII/S1	3.01			
	c.4612T>C*	p.(Trp1538Arg)	1	DIV/S2	0.20	Gain-of-function Na_v 1.7 channel	Pathogenic	[11,17]
SCN10A	c.3674T>C	p.(Ile1225Thr)	1	DIII/S3	0.055	-	VUS	[11]
	c.4568G>A	p.(Cys1523Tyr)	1	DIV/S2	0.11	DRG neuron hyperexcitability	Likely pathogenic	[9,11]
SCN11A	c.1560G>T*	p.(Gln520His)	1	Linker DI/S6-DII/S1	0.0028	-	VUS	[11]
	c.1744G>A	p.(Ala582Thr)	1	DII/S1	0.015		VUS	[11,18]
SCN4B	c.298C>T	p.(Arg100Cys)	1	β-subunit	0.0032	-	VUS	-

c.position, location cDNA; p.position, location in protein; MAF gnomAD, Minor Allele Frequency Genome Aggregation Database; VUS, Variants with uncertain clinical significance.

[&] Variants detected were annotated according to the guidelines of the Human Genome Variation Society using reference sequence GRCh37 and transcript numbers, NM_006922.3 (*SCN3A)*; NM_002977.3 (*SCN9A)*; NM_006514.2 (*SCN10A)*; NM_014139.2 (*SCN11A)*; NM_174934.4(*SCN4B).*

* One patient was heterozygous for *SCN3A* c.5583G>T, *SCN9A* c.2794A>C, c.2971G>T, *SCN9A* c.4612T>C and *SCN11A* c.1560G>T.

Supplementary Table S5. Clinical features of painful and painless diabetic peripheral neuropathy and painful and painless idiopathic small fiber neuropathy patients with and without sodium channel genes potentially pathogenic variants

	**No mutation (n=1007)**	**SCG mutation (n=118)**	**p-value**	**Painful (DPN+SFN): No mutation (n=692) vs SCG mutation (n=92)**	**p-value**	**Painless (DPN+SFN: no mutation (n=315) vs SCG mutation (n=26)**	**p-value**	**Painful-DPN: No mutation(n=209) vs SCG mutation (n=28)**	**p-value**	**Painless-DPN: No mutation (n=286) vs SCG mutation (n=23)**	**p-value**	**Painful-SFN: No mutation (n=483) vs SCG mutation (n=64)**	**p-value**	**Painless-SFN: No mutation (n=29) vs SCG mutation (n=3)**	**p-value**
**Main patient characteristics**															
Male	561/1007 (55.7%)	60/118 (50.8%)	0.315	327/692 (47.3%) vs 43/92 (46.7%)	0.926	234/315 (74.3%) vs 17/26 (65.4%)	0.322	134/209 (64.1%) vs 16/28 (57.1%)	0.472	221/286 (77.3%) vs 15/23 (65.2%)	0.190	193/483 (40.0%) vs 27/64 (42.2%)	0.733	13/29 (44.8%) vs 2/3 (66.7%)	0.589
Mean age (in years +/- SD)	59.03 (13.94) (0 missing)	57.29 (14.19) (0 missing)	0.200	56.78 (13.84) vs 55.48 (14.29)	0.399	63.98 (12.86) vs 63.69 (12.05)	0.912	64.10 (10.41) vs 60.75 (10.12)	0.110	64.96 (11.94) vs 64.43 (11.37)	0.839	53.61 (13.94) vs 53.17 (15.27)	0.815	54.34 (17.28) vs 58.00 (18.36)	0.731
Mean age of onset neuropathy (in years +/- SD)	53.31 (15.21) (378 missing)	50.32 (16.48) (50 missing)	0.128	50.11 (14.98) vs 48.48 (16.51)	0.444	60.71 (13.03) vs 61.00 (12.15)	0.944	58.30 (11.50) vs 59.75 (8.75)	0.625	62.17 (11.93) vs 64.75 (9.29)	0.548	45.77 (14.81) vs 44.19 (16.81)	0.526	45.76 (14.67) vs 46.00 (12.73)	0.983
Mean years of duration neuropathy (in years +/- SD)	7.10 (8.15) (390 missing)	6.16 (7.74) (51 missing)	0.371	7.64 (8.62) vs 6.47 (8.19)	0.329	5.78 (6.68) vs 4.22 (3.31)	0.489	7.07 (6.00) vs 3.19 (2.34)	**<0.001**	5.70 (6.72) vs 3.86 (1.95)	0.472	7.93 (9.72) vs 7.71 (9.25)	0.891	6.53 (6.38) vs 5.50 (7.78)	0.834
Familial cases of neuropathy	114/605 (18.8%)	20/60 (33.3%)	**0.008**	93/425 (21.9%) vs 17/51 (33.3%)	0.067	21/180 (11.7%) vs 3/9 (33.3%)	0.091	24/147 (16.3%) vs 6/15 (40.0%)	**0.036**	21/176 (11.9%) vs 3/9 (33.3%)	0.095	69/278 (24.8%) vs 11/36 (30.6%)	0.457	0/4 (0.00%) 0/4 (0.00%)	N/A
**NPS**															
Severity	348/472 (73.7%)	41/51 (80.4%)	0.300	295/351 (84.0%) vs 37/43 (86.0%)	0.734	53/121 (43.8%) vs 4/8 (50.0%)	0.731	113/133 (85.0%) vs 12/13 (92.3%)	0.693	50/117 (42.7%) vs 4/8 (50.0%)	0.725	182/218 (83.5%) vs 25/30 (83.3%)	1,000	3/4 (75.0%) vs ?	N/A
Sharpness	338/472 (71.6%)	38/51 (74.5%)	0.662	294/351 (83.8%) vs	0.248	44/121 (36.4%) vs	0.258	103/133 (77.4%) vs 12/13 (92.3%)	0.301	41/117 (35.0%) vs	0.143	191/218 (87.6%) vs	**0.022**	3/4 (75.0%) vs ?	N/A

				33/43 (76.7%)		5/8 (62.5%)				5/8 (62.5%)		21/30 (70.0%)			
Hotness	336/472 (71.2%)	42/51 (82.4%)	0.091	285/351 (81.2%) vs 37/43 (86.0%)	0.437	51/121 (42.1%) vs 5/8 (62.5%)	0.293	102/133 (76.7%) vs 12/13 (92.3%)	0.299	48/117 (41.0%) vs 5/8 (62.5%)	0.282	183/218 (83.9%) vs 25/30 (83.3%)	1,000	3/4 (75.0%) vs ?	N/A
Dullness	263/472 (55.7%)	30/51 (58.8%)	0.671	225/351 (64.1%) vs 28/43 (65.1%)	0.896	38/121 (31.4%) vs 2/8 (25.0%)	1,000	73/133 (54.9%) vs 9/13 (69.2%)	0.320	35/117 (29.9%) vs 2/8 (25.0%)	1,000	152/218 (69.7%) vs 19/30 (63.3%)	0.478	3/4 (75.0%) vs ?	N/A
Coldness	196/472 (41.5%)	32/51 (62.7%)	**0.004**	169/351 (48.1%) vs 28/43 (65.1%)	**0.036**	27/121 (22.3%) vs 4/8 (50.0%)	0.094	61/133 (45.9%) vs 9/13 (69.2%)	0.107	25/117 (21.4%) vs 4/8 (50.0%)	0.083	108/218 (49.5%) vs 19/30 (63.3%)	0.157	2/4 (50.0%) vs ?	N/A
Sensitivity	241/472 (51.1%)	37/51 (72.5%)	**0.003**	217/351 (61.8%) vs 33/43 (76.7%)	0.055	24/121 (19.8%) vs 4/8 (50.0%)	0.067	64/133 (48.1%) vs 9/13 (69.2%)	0.146	22/117 (18.8%) vs 4/8 (50.0%)	0.058	153/218 (70.2%) vs 24/30 (80.0%)	0.265	2/4 (50.0%) vs ?	N/A
Itchiness	171/472 (36.2%)	23/51 (45.1%)	0.213	147/351 (41.9%) vs 21/43 (48.8%)	0.384	24/121 (19.8%) vs 2/8 (25.0%)	0.662	58/133 (43.6%) vs 7/13 (53.8%)	0.478	22/117 (18.8%) vs 2/8 (25.0%)	0.649	89/218 (40.8%) vs 14/30 (46.7%)	0.543	2/4 (50.0%) vs ?	N/A
Unpleasantness	375/471 (79.5%)	47/51 (92.2%)	**0.031**	317/350 (90.6%) vs 39/43 (90.7%)	1,000	58/121 (47.9%) vs 8/8 (100%)	**0.006**	119/133 (89.5%) vs 12/13 (92.3%)	1,000	55/117 (47.0%) vs 8/8 (100%)	**0.006**	198/217 (91.2%) vs 27/30 (90.0%)	0.737	3/4 (75.0%) vs ?	N/A
Intensity deep pain	340/471 (72.2%)	40/51 (78.4%)	0.341	287/350 (82.0%) vs 34/43 (79.1%)	0.639	53/121 (43.8%) vs 6/8 (75.0%)	0.141	102/133 (76.7%) vs 9/13 (69.2%)	0.512	50/117 (42.7%) vs 6/8 (75.0%)	0.138	185/217 (85.3%) vs 25/30 (83.3%)	0.786	3/4 (75.0%) vs ?	N/A
Intensity surface pain	313/471 (66.5%)	40/51 (78.4%)	0.082	263/350 (75.1%) vs 36/43 (83.7%)	0.213	50/121 (41.3%) vs 4/8 (50.0%)	0.719	90/133 (67.7%) vs 10/13 (76.9%)	0.755	47/117 (40.2%) vs 4/8 (50.0%)	0.715	173/217 (79.7%) vs 26/30 (86.7%)	0.368	3/4 (75.0%) vs ?	N/A
**SFN-SIQ**															
Altered sweating pattern	397/538 (73.8%)	40/56 (71.4%)	0.703	302/370 (81.6%) vs 38/48 (79.2%)	0.681	95/168 (56.5%) vs 2/8 (25.0%)	0.142	100/138 (72.5%) vs 10/14 (71.4%)	1,000	91/164 (55.5%) vs 2/8 (25.0%)	0.145	202/232 (87.1%) vs 28/34 (82.4%)	0.427	4/4 (100%) vs ?	N/A
Diarrhea	309/538 (57.4%)	34/56 (60.7%)	0.636	225/370 (60.8%) vs	0.821	84/168 (50.0%) vs	1,000	78/138 (56.5%) vs 8/14 (57.1%)	0.964	80/164 (48.8%) vs	1,000	147/232 (63.4%) vs	0.879	4/4 (100%) vs ?	N/A

				30/48 (62.5%)		4/8 (50.0%)				4/8 (50.0%)		22/34 (64.7%)			
Constipation	299/537 (55.7%)	34/56 (60.7%)	0.470	230/369 (62.3%) vs 30/48 (62.5%)	0.982	69/168 (41.1%) vs 4/8 (50.0%)	0.720	78/138 (56.5%) vs 8/14 (57.1%)	0.964	66/164 (40.2%) vs 4/8 (50.0%)	0.717	152/231 (65.8%) vs 22/34 (64.7%)	0.900	3/4 (75.0%) vs ?	N/A
Micturition problems	329/538 (61.2%)	38/56 (67.9%)	0.326	246/370 (66.5%) vs 36/48 (75.0%)	0.236	83/168 (49.4%) vs 2/8 (25.0%)	0.280	75/138 (54.3%) vs 9/14 (64.3%)	0.476	80/164 (48.8%) vs 2/8 (25.0%)	0.282	171/232 (73.7%) vs 27/34 (79.4%)	0.476	3/4 (75.0%) vs ?	N/A
Dry eyes	340/538 (63.2%)	47/56 (83.9%)	**0.002**	259/370 (70.7%) vs 40/48 (83.3%)	0.054	81/168 (48.2%) vs 7/8 (87.5%)	0.064	85/138 (61.6%) vs 9/14 (64.3%)	0.843	78/164 (47.6%) vs 7/8 (87.5%)	**0.033**	174/232 (75.0%) vs 31/34 (91.2%)	**0.036**	3/4 (75.0%) vs ?	N/A
Dry mouth	391/538 (72.7%)	47/56 (83.9%)	0.069	305/370 (82.4%) vs 40/48 (83.3%)	0.877	86/168 (51.2%) vs 7/8 (87.5%)	0.068	100/138 (72.5%) vs 10/14 (71.4%)	1,000	83/164 (50.6%) vs 7/8 (87.5%)	0.066	205/232 (88.4%) vs 30/34 (88.2%)	1,000	3/4 (75.0%) vs ?	N/A
Orthostatic dizziness	362/536 (67.5%)	42/56 (75.0%)	0.254	278/368 (75.5%) vs 38/48 (79.2%)	0.581	84/168 (50.0%) vs 4/8 (50.0%)	1,000	103/137 (75.2%) vs 12/14 (85.7%)	0.520	80/164 (48.8%) vs 4/8 (50.0%)	1,000	175/231 (75.8%) vs 26/34 (76.5%)	0.928	4/4 (100%) vs ?	N/A
Palpitations	307/536 (57.3%)	35/56 (62.5%)	0.451	237/368 (64.4%) vs 30/48 (62.5%)	0.796	70/168 (41.7%) vs 5/8 (62.5%)	0.288	87/137 (63.5%) vs 7/14 (50.0%)	0.321	66/164 (40.2%) vs 5/8 (62.5%)	0.277	150/231 (64.9%) vs 23/34 (67.6%)	0.756	4/4 (100%) vs ?	N/A
Hot flashes	253/536 (47.2%)	40/56 (71.4%)	**0.001**	222/368 (60.3%) vs 37/48 (77.1%)	**0.024**	31/168 (18.5%) vs 3/8 (37.5%)	0.184	47/137 (34.3%) vs 7/14 (50.0%)	0.243	29/164 (17.7%) vs 3/8 (37.5%)	0.169	175/231 (75.8%) vs 30/34 (88.2%)	0.105	2/4 (50.0%) vs ?	N/A
Sensitive skin legs	399/536 (74.4%)	45/56 (80.4%)	0.331	302/368 (82.1%) vs 39/48 (81.3%)	0.890	97/168 (57.7%) vs 6/8 (75.0%)	0.472	106/137 (77.4%) vs 8/14 (57.1%)	0.108	95/164 (57.9%) vs 6/8 (75.0%)	0.473	196/231 (84.8%) vs 31/34 (91.2%)	0.437	2/4 (50.0%) vs ?	N/A
Burning feet	457/536 (85.3%)	52/56 (92.9%)	0.119	347/368 (94.3%) vs 46/48 (95.8%)	1,000	110/168 (65.5%) vs 6/8 (75.0%)	0.718	127/137 (92.7%) vs 13/14 (92.9%)	1,000	108/164 (65.9%) vs 6/8 (75.0%)	0.719	220/231 (95.2%) vs 33/34 (97.1%)	1,000	2/4 (50.0%) vs ?	N/A
Intolerance for sheets	304/536 (56.7%)	36/56 (64.3%)	0.276	270/368 (73.4%) vs 33/48 (68.8%)	0.499	34/168 (20.2%) vs 3/8 (37.5%)	0.367	79/137 (57.7%) vs 8/14 (57.1%)	0.970	30/164 (18.3%) vs 3/8 (37.5)	0.181	191/231 (82.7%) vs 25/34 (73.5%)	0.199	4/4 (100%) vs ?	N/A

Restless legs	368/536 (68.7%)	46/56 (82.1%)	**0.036**	300/368 (81.5%) vs 44/48 (91.7%)	0.081	68/168 (40.5%) vs 2/8 (25.0%)	0.480	99/137 (72.3%) vs 12/14 (85.7%)	0.356	64/164 (39.0%) vs 2/8 (25.0%)	0.712	201/231 (87.0%) vs 32/34 (94.1%)	0.395	4/4 (100%) vs ?	N/A

Painful-DPN, painful diabetic peripheral neuropathy; Painless-DPN, painless diabetic peripheral neuropathy; Painful-SFN, painful-idiopathic small fiber neuropathy; Painless-SFN, painless-idiopathic small fiber neuropathy; SCG, sodium channel genes; mutation, potentially pathogenic variant; NPS; Neuropathic Pain Scale; SFN-SIQ, SFN symptoms inventory questionnaire; n, number; SD, standard deviation; N/A, not applicable.

A

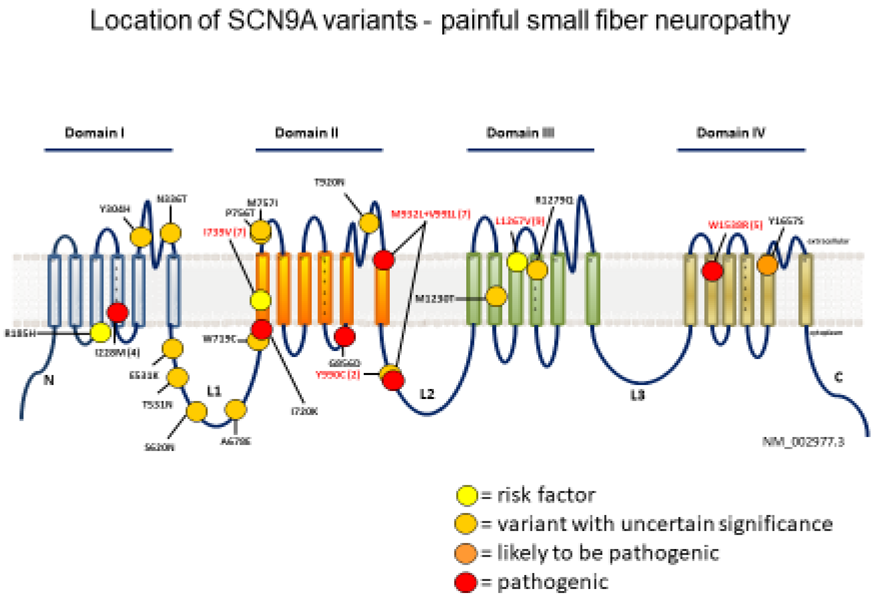

PROPANE study cohort (n=547 pat)

B

Location of SCN9A variants - painful diabetic neuropathy

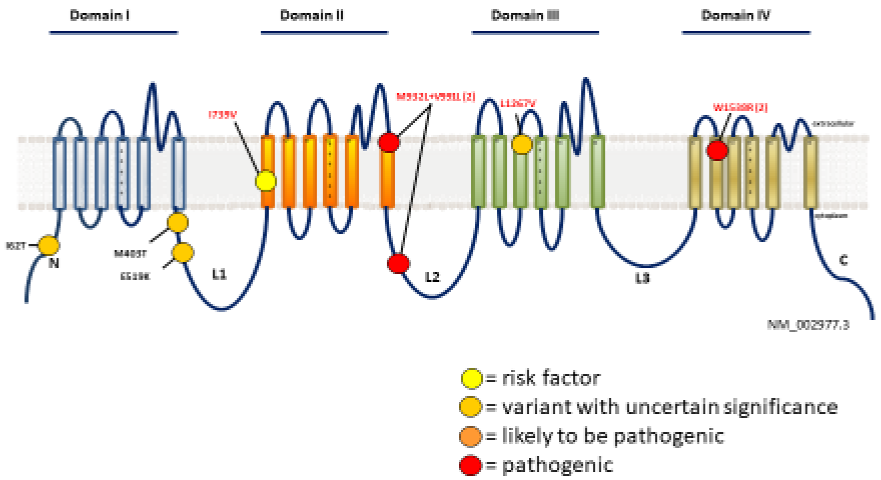

PROPANE study cohort (n=237 pat)

C

Location of SCN9A variants - painless diabetic neuropathy

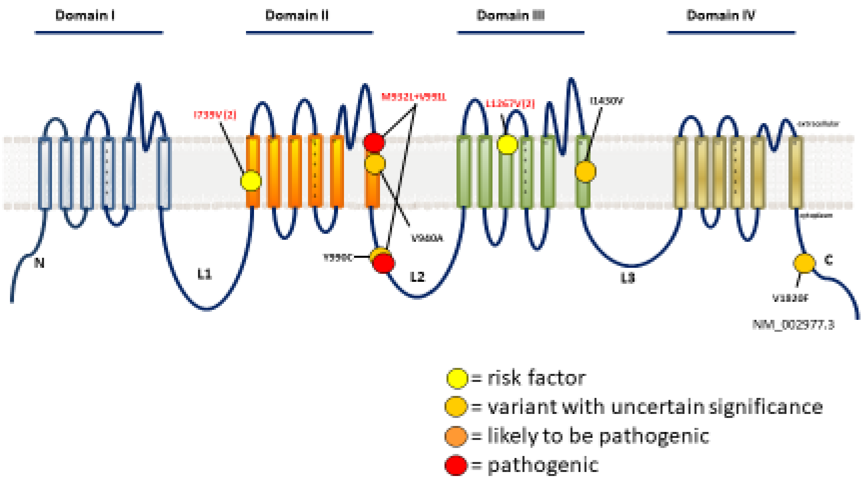

PROPANE study cohort (n=309 pat)

D

Location of SCN10A variants - painful small fiber neuropathy

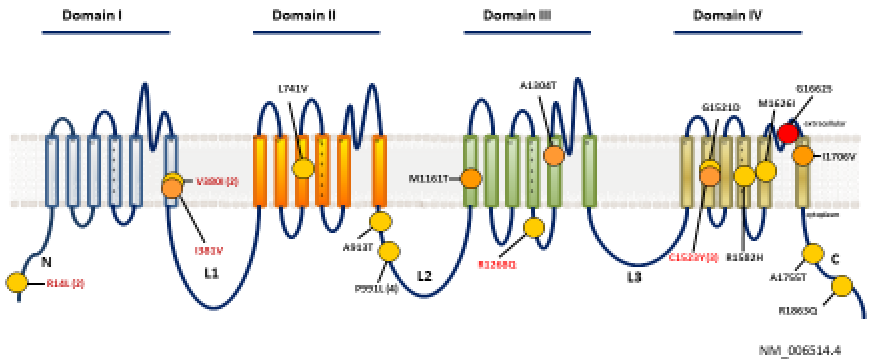

= Class 3, variant with uncertain significance
= Class 4, likely to be pathogenic
= Class 5, pathogenic

PROPANE study cohort (n=547 pat)

E

Location of SCN10A variants - painless diabetic neuropathy

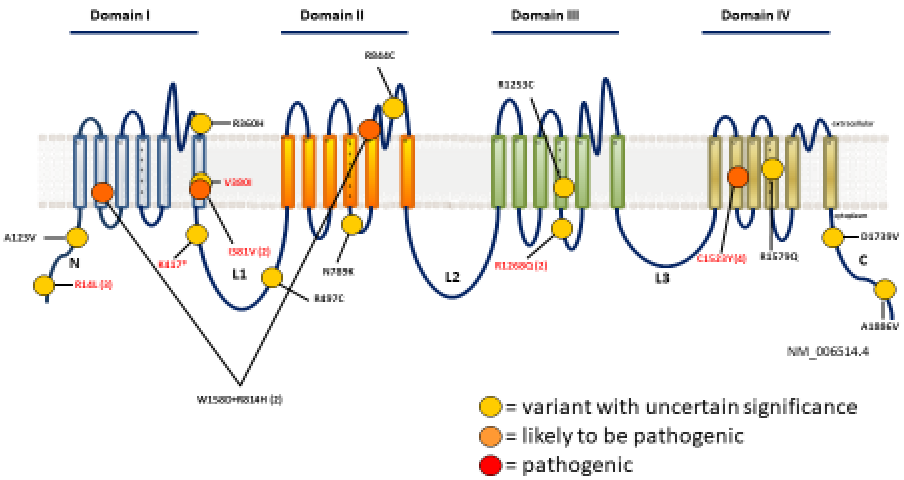

PROPANE study cohort (n=309 pat)

F

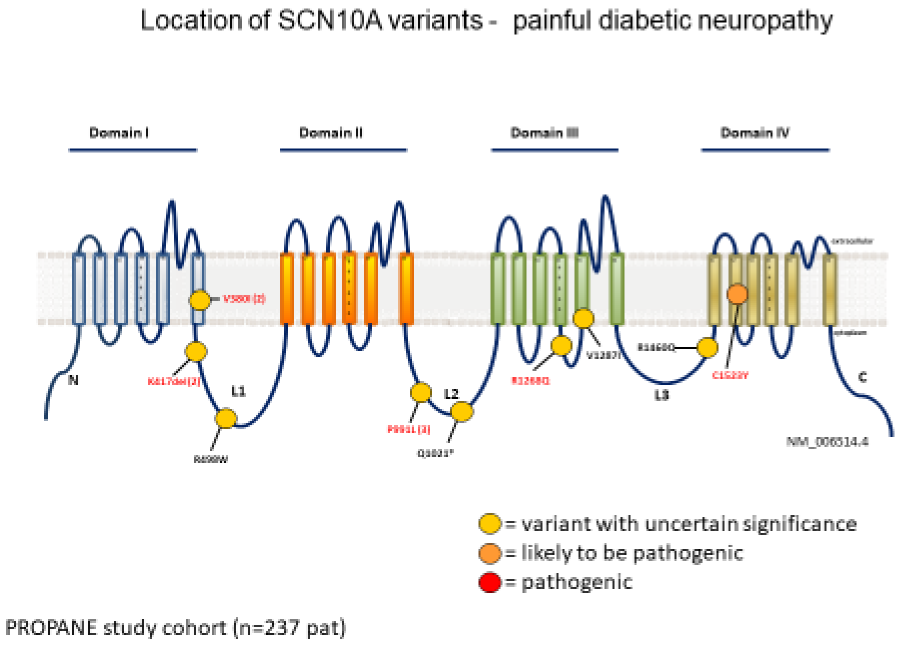

Supplementary Figure S2. SCG variants distribution in painful and painless DPN and SFN patients. (A, B, and C) shows the distribution of *SCN9A* variants in painful SFN, painful-DPN, and painless-DPN patients respectively. The distribution of *SCN10A* variants in painful SFN, painful-DPN, and painless-DPN patients is shown in (D, E, and F).
